# Elimination of HIV-1 Genomes from Human T-lymphoid Cells by CRISPR/Cas9 Gene Editing

**DOI:** 10.1038/srep22555

**Published:** 2016-03-04

**Authors:** Rafal Kaminski, Yilan Chen, Tracy Fischer, Ellen Tedaldi, Alessandro Napoli, Yonggang Zhang, Jonathan Karn, Wenhui Hu, Kamel Khalili

**Affiliations:** 1 Department of Neuroscience/Center for Neurovirology, Lewis Katz School of Medicine at Temple University, 3500 N. Broad Street, 7th Floor, Philadelphia, PA 19140 USA; 2 Comprehensive NeuroAIDS Center, Lewis Katz School of Medicine at Temple University, 3500 N. Broad Street, 7th Floor, Philadelphia, PA 19140 USA; 3 Department of Medicine, Temple HIV Program, Lewis Katz School of Medicine at Temple University/Temple University Hospital, 3400 N. Broad Street, Philadelphia, PA 19140 USA; 4 Department of Molecular Biology and Microbiology, Case Western Reserve University, Cleveland, OH 44106, USA

## Abstract

We employed an RNA-guided CRISPR/Cas9 DNA editing system to precisely remove the entire HIV-1 genome spanning between 5′ and 3′ LTRs of integrated HIV-1 proviral DNA copies from latently infected human CD4+ T-cells. Comprehensive assessment of whole-genome sequencing of HIV-1 eradicated cells ruled out any off-target effects by our CRISPR/Cas9 technology that might compromise the integrity of the host genome and further showed no effect on several cell health indices including viability, cell cycle and apoptosis. Persistent co-expression of Cas9 and the specific targeting guide RNAs in HIV-1-eradicated T-cells protected them against new infection by HIV-1. Lentivirus-delivered CRISPR/Cas9 significantly diminished HIV-1 replication in infected primary CD4+ T-cell cultures and drastically reduced viral load in *ex vivo* culture of CD4+ T-cells obtained from HIV-1 infected patients. Thus, gene editing using CRISPR/Cas9 may provide a new therapeutic path for eliminating HIV-1 DNA from CD4+ T-cells and potentially serve as a novel and effective platform toward curing AIDS.

AIDS remains a major public health problem, as over 35 million people worldwide are HIV-1-infected and new infections continue at steady rate of greater than two million per year. Antiretroviral therapy (ART) effectively controls viremia in virtually all HIV-1 patients and partially restores the primary host cell (CD4+ T-cells), but fails to eliminate HIV-1 from latently-infected T-cells^1^,^2^. In latently-infected CD4+ T cells, integrated proviral DNA copies persist in a dormant state, but can be reactivated to produce replication-competent virus when T-cells are activated, resulting in rapid viral rebound upon interruption of antiretroviral treatment^3^,^4^,^5^,^6^,^7^,^8^. Therefore, most HIV-1-infected individuals, even those who respond very well to ART, must maintain life-long ART due to persistence of HIV-1-infected reservoir cells. During latency HIV infected cells produce little or no viral protein, thereby avoiding viral cytopathic effects and evading clearance by the host immune system. Because the resting CD4+ memory T-cell compartment^9^ is thought to be the most prominent latently-infected cell pool, it is a key focus of research aimed at eradicating latent HIV-1 infection.

Recent efforts to eradicate HIV-1 from this cell population have used primarily a “shock and kill” approach, with the rationale that inducing HIV reactivation in CD4+ memory T-cells may trigger elimination of virus-producing cells by cytolysis or host immune responses. For example, epigenetic modification of chromatin structure is critical for establishing viral reactivation. Consequently, inhibition of histone deacetylase (HDAC) by Trichostatin A (TSA) and vorinostat (SAHA) led to reactivation of latent virus in cell lines^10^,^11^,^12^. Accordingly, other HDACi, including vorinostat, valproic acid, panobinostat and rombidepsin have been tested *ex vivo* and have led, in the best cases, to transient increases in viremia^13^,^14^. Similarly, protein kinase C agonists, can potently reactivate HIV either singly or in combination with HDACi^15^,^16^. However, there are multiple limitations of this approach: (i) since a large fraction of HIV genomes in this reservoir are non-functional, not all integrated provirus can produce replication-competent virus^17^; (ii) total numbers of CD4+ T cells reactivated from resting CD4+ T cell HIV-1 reservoirs, has been found by viral outgrowth assays to be much smaller than the numbers of cells infected, as detected by PCR-based assays, suggesting that not all cells within this reservoir are reactivated^18^; (iii) the cytotoxic T lymphocyte (CTL) immune response is not sufficiently robust to eliminate the reactivated infected cells^19^ and (iv) uninfected T-cells are not protected from HIV infection and can therefore sustain viral rebound.

These observations suggest that a cure strategy for HIV-1 infection should include methods that directly eliminate the proviral genome from the majority of HIV-1-positive cells, including CD4+ T-cells, and protect cells from future infection, with little or no harm to the host. The clustered, regularly-interspaced, short palindromic repeats (CRISPR)/CRISPR-associated 9 (Cas9) nuclease has wide utility for genome editing in a broad range of organisms including yeast, *Drosophila*, zebrafish, *C. elegans*, and mice, and has been applied in a broad range of *in vivo* and *in vitro* studies toward human diseases^20^,^21^,^22^,^23^,^24^. Recently we modified the CRISPR/Cas9 system to enable recognition of specific DNA sequences positioned within the HIV-1 promoter spanning the 5′ long terminal sequence (LTR)^25^,^26^. Using this modified system, we now demonstrate excision of integrated copies of the proviral DNA fragment from a latently HIV-1-infected human T-lymphoid cell line, completely eliminating HDAC inhibition-elicited viral production. Results of whole-genome sequencing and comprehensive bioinformatic analysis ruled out any genotoxicity to host cell DNA. Further, we found that lentivirally-delivered CRISPR/Cas9 reduces viral replication upon HIV-1 infection of primary cultured CD4+ T-cells. The results point toward this approach as a promising potential therapeutic avenue to eradicating HIV-1 from T reservoir cells of host patients, to prevent AIDS re-emergence.

## Results

### Cas9/gRNA inhibits HIV-1 reactivation of latent HIV-1 in human T-cells

**Figure 1:**
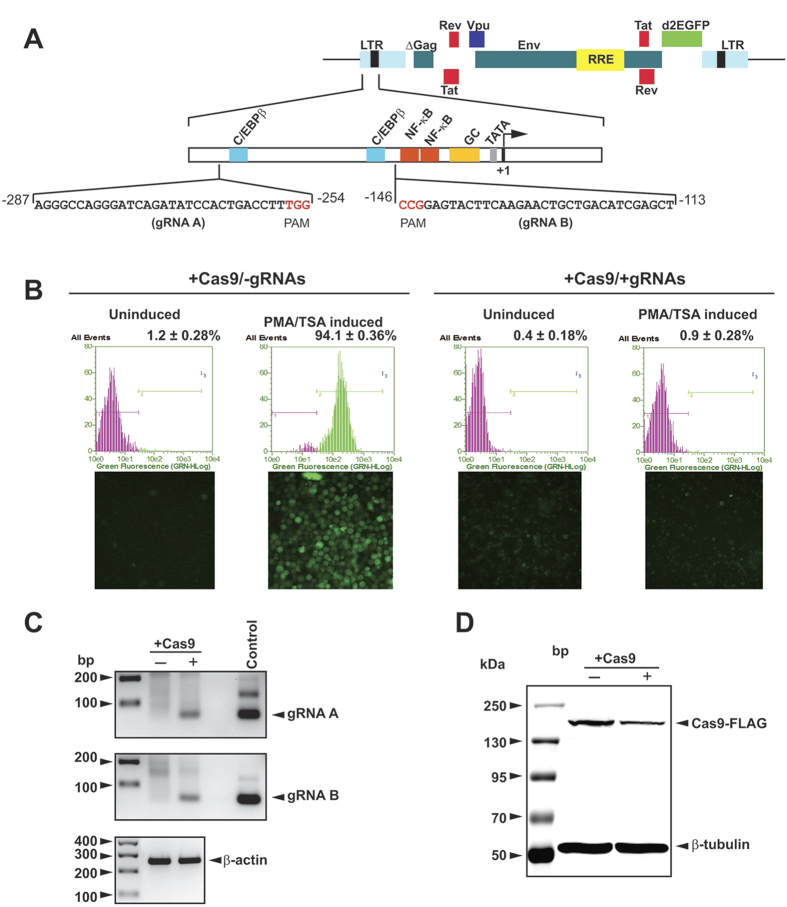
CRISPR/Cas9 eliminates HIV-1 expression in PMA/TSA treated, latently-infected human T-cell line. **(A)** (*Top*) Schematic representation of the structural organization of the integrated HIV-1 proviral DNA highlighting the position of the long terminal repeat (LTR), various viral genes spanned by the LTR, and the location of the reporter d2EGFP. (*Bottom*) Illustration of the 5′ LTR and the nucleotide sequences of target regions A (gRNA A) and B (gRNA B) used for editing, and the motifs for binding of the various transcription factors. Arrow at +1 depicts the transcription start site. (**B)** Gating diagram of EGFP flow cytometry and fluorescence microscopic imaging of the CD4+ T-cells before and after PMA/TSA treatment shows PMA/TSA-induced reactivation of latent virus in control cells expressing only Cas9, but not in cells expressing both Cas9 and gRNA. (**C)** RT-PCR-based detection of gRNA A, gRNA B and β-actin RNA in cells transfected with plasmids expressing Cas9 ± gRNAs. β-actin is the RNA loading control. (**D)** Detection of Cas9 protein by Western blot analysis in control cells and cells with ablated HIV-1/EGFP expression. β-tubulin served as the protein loading control.

We first tested the ability of our modified CRISPR/Cas9 gene editing system to eliminate the HIV-1 genome in a human T-lymphocytic cell line, 2D10^11^. These cells harbor integrated copies of a single round HIV-1_PNL4-3_ whose genome lacks sequences encoding the majority of the Gag-Pol polyprotein, but encompasses the full-length 5′ and 3′ LTRs, and includes a gene encoding the marker protein green fluorescent protein (GFP) replacing Nef protein in the latent state (Fig. 1A). Thus, 2D10 is a suitable cell line to first establish proof-of-principle of HIV-1 eradication because of the uniform nature of the integrated provirus. Treatment of clonal 2D10 cells stably expressing Cas9, but not gRNAs, with proinflammatory agents such as phorbol myristate acetate (PMA) and/or the HDAC inhibitor trichostatin A (TSA) profoundly stimulates HIV-1 promoter activity, leading to production of the viral proteins and GFP in over 90% of treated cells (Fig. 1B, left panels), providing a convenient cell culture model for studying viral latency and reactivation. Co-expression of Cas9 along with gRNAs A and B, designed respectively to target the highly conserved sequence among all viral isolates spanning the LTR U3 region at nt −287/−254 (gRNA A) and nt −146/−113 (gRNA B) (Fig. 1A) completely eliminated PMA/TSA-induced GFP production, indicating inhibition of HIV-1 gene expression in the pre-selected mixed clonal population of T-cells expressing both Cas9 and gRNA expression plasmids (Fig. 1B, right panels). Expression of gRNAs and Cas9 was verified by RT-PCR and Western blot, respectively (Fig. 1C,D). HIV-1 expression was completely eliminated from the cells expressing both Cas9 and gRNA expression plasmids, shown by flow cytometry detection of GFP production by randomly-selected Cas9-positive clonal cells with or without gRNA expression (Fig. S2A). Also, we found that GFP production was effectively blocked in many clones that expressed only a single gRNA (A or B), to levels similar to those elicited by co-expression of both A and B (Fig. S2B; also see Fig. S2A), suggesting that expression of either gRNA in single configuration can initiate cleavage at both LTRs to achieve eradication of proviral DNA.

### Integration sites of HIV-1 proviral DNA in human T-cells and excision of viral DNAs from host cell chromosomes

We verified the site(s) of HIV–1 proviral DNA integration by whole–genome sequencing (WGS) of 2D10 cells. We used CREST calling^27^ of the structural variation (SV) to identify breakpoints caused by proviral DNA integration in the host genome, and used the hg19 genome and the HIV–1 genome, KM390026.1 as reference genomes for reading the DNA sequences. We identified four inter–chromosomal translocations, designated by CTX (Fig. S3), that are related to HIV-1 DNA. Breakpoints between the HIV-1 5′ LTR and P163.3:1991382 and the HIV-1 3′ LTR P613.3:1991378 were detected, mapping to exon 2 of the methionine sulfoxide reductase B1 MSRB1 gene (NM_01332), and corresponding to a previously mapped location for the provirus in the 2D10 cells^11^,^28^. In addition, two CTXs were mapped to chromosome 1 with the breakpoint between P13.2:114338315 and the HIV-1 5′ LTR, and other breakpoints between HIV-1 3′ LTR and P13.2:114338320. Also, we noted that four nucleotides, TAAG, were deleted between the two breakpoints in chromosome 1P13.2. The HIV-1 provirus in chromosome 1, which was previously undetected by linker-addition mapping, was integrated in the second intron (114339984–114320431) of the round spermatid basic protein 1 (RSBN1) gene (NM_018364). A schematic presentation of identified consensus sequences for sites of HIV-1 DNA integration in chromosomes 1 and 16 are shown in Fig. S3.

**Figure 2:**
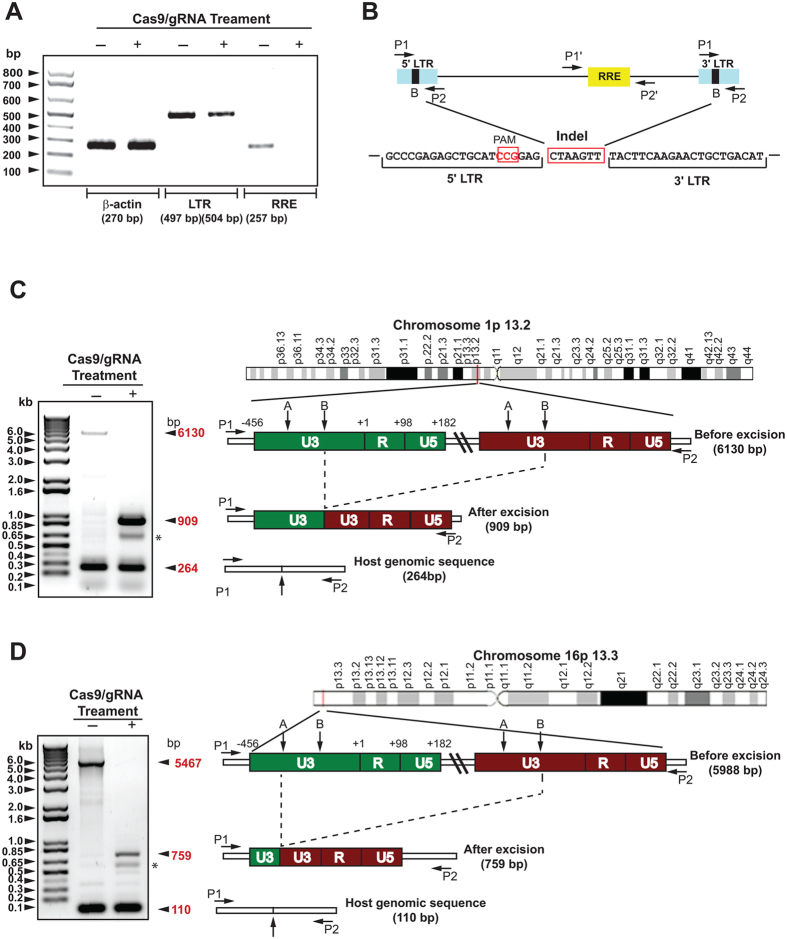
Elimination of integrated HIV-1 DNA from the host T cell genome by Cas9/gRNAs targeting viral LTRs. (**A)** DNA analysis shows 497- and 504-nucleotide amplicons detected, corresponding respectively to the HIV-1 LTRs in control cells and in cells co-expressing Cas9 and gRNAs. Positions of the amplicons corresponding to the RRE and β-actin are shown. (**B)** Nucleotide composition of the amplified LTR DNA from CRISPR/Cas9-treated cells along with the positions of primers used for PCR amplification of the various regions of the viral genome. Integration of the 7-nucleotide InDel mutation after removal of the viral DNA fragment positioned between the B-motif of the 5′ and 3′ LTRs is shown. The seed sequence for gRNA B is highlighted in black. (**C,D)** The sites of HIV-1 integration in Chromosome 1 (Panel **C**) and Chromosome 16 (Panel **D**) are shown. In each panel, results of DNA analysis of the PCR product amplified by the specific primers (P1 and P2) derived from the cellular genes interrupted by viral DNA insertions are shown. Diagrams of each chromosome containing full-length integrated HIV-1 DNA before CRISPR/Cas9 treatment and the residual LTR DNA sequence after Cas9/gRNAs treatment are depicted, based on Sanger sequencing of the major DNA fragments seen on agarose gel. The asterisks in Panels **C** and **D** point to the minor DNA bands indicating the complete removal of viral DNA when either A or B targets within the 5′ or 3′ LTRs were used.

Short-range amplification assay of LTR DNA revealed an expected 497-bp DNA fragment in control cells and a second DNA fragment of similar size (504 bp) after treatment with Cas9/gRNAs A and B (Fig. 2A). Results of direct DNA sequencing of the PCR amplicon suggested that the observed 504-bp DNA fragment in Cas9/gRNA-treated cells was created by joining of the residual 5′ LTR to the remaining 3′ LTR after cleavage by Cas9/gRNA B (Fig. 2B). An Indel mutation with a seven-nucleotide insertion was also detected in the junction of the 5′ and 3′ fusion site of the clonal cells (Fig. 2B). The 257-bp PCR amplicon corresponding to the Rev response element (RRE), which is positioned in the center of the viral genome, was absent, verifying that Cas9/gRNAB removed the DNA sequences spanning between the two terminal repeats (Fig. 2A). Long-range PCR analysis of 2D10 control cells expressing Cas9 but not gRNAs, using a pair of primers derived from the second intron of RSBN1, verified the presence of a 6130-bp DNA fragment corresponding to the integrated HIV-1 genome plus its chromosome 1-derived flanking DNA sequence (Fig. 2C). The 264-nucleotide DNA fragment that represents host cell DNA sequence from the other copy of chromosome 1 was also present (shown at the bottom of the gel). In cells treated with Cas9/gRNAs A and B, a DNA fragment of 6130 nucleotides corresponding to the integrated HIV-1 genome was completely absent. Instead, PCR amplification produced a smaller DNA fragment of 909 nucleotides. Sequencing of the amplicon verified excision of the integrated viral DNA, spanning between the B domain of the 5′ LTR and the B domain of the 3′ LTR (Fig. S4A,B). Again, we detected a 264-nucleotide DNA fragment amplified from the host genome from the other chromosome (Fig. 2C).

We examined chromosome 16 for presence of HIV-1 proviral DNA using long-range PCR using a primer pair corresponding to the second exon of MSRB1 gene and compared its status in Cas9/gRNA A/B-treated cells. The results showed that the expected 5467-bp DNA fragment of the HIV-1 genome and its flanking host DNA in chromosome 16 was absent. Instead we detected a smaller 759-bp DNA fragment that reflected joining of the residual U3 region of the 5′ LTR after cleavage by gRNA A to the remaining U3 region of the 3′ LTR upon cleavage by gRNA B (Fig. 2D). Direct sequencing of the 759-bp DNA fragment identified the sites of viral DNA excision (Fig. S5A,B). A smaller, 110-bp DNA fragment found resulted from amplification of host DNA from the other copy of chromosome 16. These observations provide strong evidence that the gene editing molecules used effectively eliminate multiple copies of the integrated proviral DNA of the HIV-1-genome, which are scattered among various chromosomes.

### Elimination from host cells of HIV-1 DNA sequence spanning between 5′ and 3′ LTRs, and positions of the breakpoints

**Figure 3:**
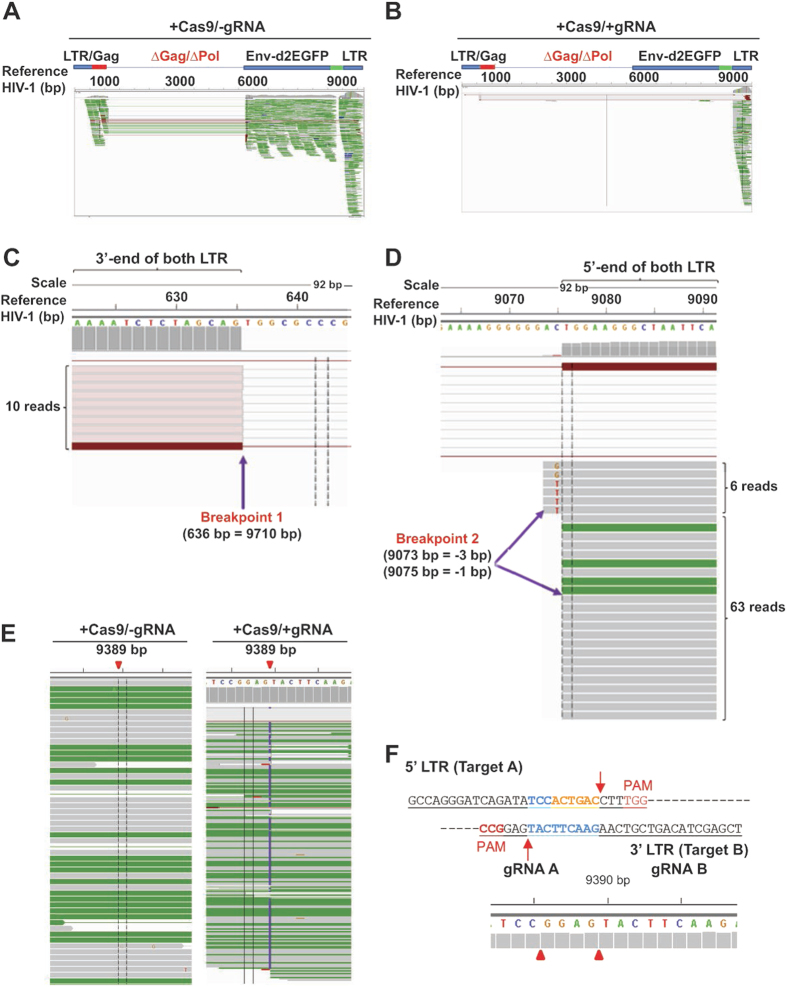
Whole-genome sequencing shows excision of the entire provirus of two copies of HIV-1 by Cas9/gRNAs and gRNAs A and B in human T cells. (**A**,**B**) Integrative genomics view of the reads mapping against the HIV-1 genome (KM390026.1) called by BWA, revealed the presence of the HIV-1 proviral DNA sequence in the control cells with expression of Cas9 but not gRNAs (Panel **A**) but their complete absence in T-cells after expression of both Cas9 and gRNAs A and B (Panel **B**). (**C,D**) Structural variant CREST analysis identifies two breakpoints at the 5′ and 3′ ends of both LTRS supported by indicated reads after cleavage of Cas9/gRNAs A/B. Integrative genomics view (IGV) of the reads mapping against HIV-1 genome (KM390026.1) is illustrated. **(E)** Identification of gRNAs (**A,B**) specific breakpoint at 9389 site (red arrowhead) by structural variants called by CREST. The vertical purple line points to the position where the remaining of the 5′ and 3′ LTRs after cleavage were joined. **(F)** Illustration of DNA sequence at the junction site (red arrowhead) after removal of the nucleotides between the precise cut sites, i.e. three nucleotides from PAM (red arrow) of the 5′ LTR at target A by gRNA A and the 3′ LTR at target B by gRNA B.

To further validate the efficiency of our Cas9/gRNA treatment–based gene editing strategy in eliminating HIV– 1 proviral DNA from latently–infected T–cells, we analyzed the occurrence of insertion/deletion (InDel) and single nucleotide polymorphisms (SNP) in the HIV–1 genomes of control and HIV–1 –eradicated cells, using GATK calling^29^ against reference HIV–1 DNA (GenBank accession #KM3900261). Consistent with the results shown in Fig. 2, the reads from the whole-genome sequencing mapped to the 5′- and 3′-LTRs and to the proviral genome in the control 2D10 cells, supporting the precision and reliability of the deep coverage of the HIV-1 DNA by genome sequencing (Fig. 3A). In Cas9/gRNA-treated 2D10 cells, the integrated genomics view (IGV) revealed complete removal of a large DNA fragment corresponding to the HIV-1 proviral DNA with reads that map to the 3′ LTR (Fig. 3B). Reads mapping to the entire proviral genome between the two LTRs were completely absent, suggesting that both copies of the integrated HIV-1 genome in host cells were fully eliminated, and that Cas9/gRNA expression in a single clonal cell can attain 100% gene editing/elimination, perhaps attributable to repeated genome editing by stably-expressed Cas9/gRNA. Further, these results suggest that, after cleavage of viral 5′ and 3′ LTRs and excision of the viral genome, viral DNA is likely degraded, such that no transposition or re-integration occurs into the host genome.

To determine the repair events after Cas9/gRNA A/B–induced cleavage of both LTRs, we used BWA calling^27^ of the structural variant (SV) in the DNA from cells with HIV–1 excision, and identified the breakpoints of large insertions and/or deletions. The results verified that no excised HIV–1 DNA from one chromosome was inserted in the host genome and/or in the integrated copy of proviral DNA on the other chromosome further ruling out the notion of re-integration of the excised viral DNA into host cell genome. However, we identified three breakpoints caused by deletion of the DNA fragments corresponding to sites of viral DNA integration into the host genome. One left breakpoint positioned at the end of the 5′ LTR at nucleotide 636 (=HIV: 9710) as supported by 10 reads. One right breakpoint exhibited two patterns, one at HIV: 9073 (=HIV:-3) supported by 6 reads with 2 C → G and 4 C → T conversions; and HIV: 9075 (=HIV:-1) supported by 63 reads (Fig. 3C,D). Of note, these two breakpoints can actually reflect the presence of the entire 634 nucleotides of the LTR after the excision of full proviral DNA by Cas9/gRNAs A or B at the 5′ and 3′ LTRs followed by precise rejoining of the DNA at the cleavage site. A third breakpoint is located in the middle of the 3′ LTR at nucleotide 9389 (=HIV: 313) with C insertion supported by 87/161 reads and CTAAGTT insertion supported by 69/161 reads (Fig. 3E). This breakpoint represents the joining of DNA after cleavage at sites A and B of the 5′ and 3′ LTRs (Fig. 3F).

### Effect of excision of HIV-1 proviral DNA on the neighboring gene expression and off-target effects

**Figure 4:**
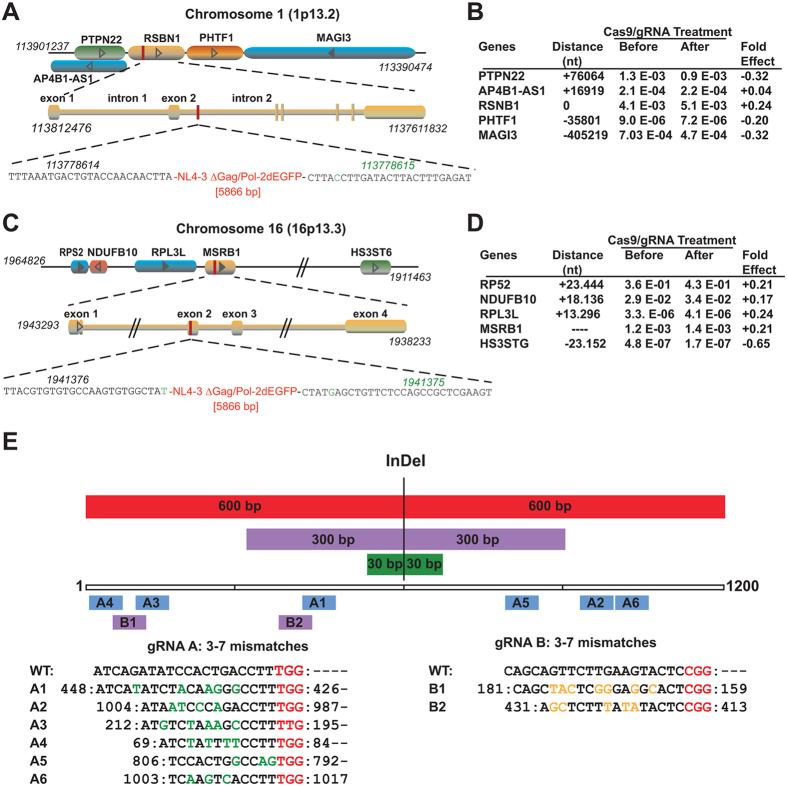
Impact of HIV-1-directed gene editing strategy on the host gene. (**A)** Schematic presentation of Chromosome 1 highlighting the site of integration of HIV-1 proviral DNA in the cellular gene, RSBN1, and the position of neighboring genes. (**B)** Expression of genes positioned at various proximities to the site of proviral integration before and after excision of the viral DNA by Cas9/gRNAs. Expression of the genes was identified by reverse transcription and qPCR, and the values were normalized to β-actin transcript. (**C)** Linear structural organization of a segment of Chromosome 16 illustrating the position of MSRB1, the site of HIV-1 DNA integration and the nucleotide structure of exon 2 of MSRB1 where viral DNA is inserted. The position of several cellular genes near MSRB1 are shown. (**D)** Results from SyberGreen qPCR illustrating expression of MSRB1 and it neighboring gene expression in cells prior to HIV-1 DNA eradication and after DNA eradication. The table shows target/reference for each cellular gene transcript obtained from 5 separate control and 5 separate HIV-1 eradicated single cell clones. (**E)** Off-target evaluation by whole genome sequencing and bioinformatic interpretation. Graphic diagram demonstrates the position of predicted off-target sites with 3–7 nucleotide mismatches within the expanded 30, 300 and 600 bp flanking the filtered InDel sites in T-cells with excised HIV-1 DNA. The numbers beside the off-target sequence indicate the nucleotides of the 1200 bp expansion sequence. The mismatched nucleotides were highlighted in green in gRNA A off-target sites (blue) and orange in gRNA B off-target sites (purple). The PAM sequence was underlined with red. Of note, the off-target locations are far from the position of the InDels and exhibit no mutations at the predicted third nucleotide from PAM.

We also investigated the impact of CRISPR/Cas9-mediated excision of HIV-1 proviral DNA from the RSBN1 gene on the level of RNA production from RSBN1 and several of the other cellular genes positioned in close proximity of the proviral insertion sites, as shown in Fig. 4A. Results from RT-PCR of five controls and five HIV-1 eradicated single cell clones indicated no significant effect on the level of expression of RSBN1, although smaller variations of less than 0.4-fold were detected in the levels of neighboring RNA (Fig. 4B), which may not be attributed to the gene editing strategy and may not impact the overall expression of their proteins. Similarly, elimination of the HIV-1 genome from chromosome 16 showed no significant impact on the expression of the site of integration, i.e. MSRB1 gene and its surrounding gene (Fig. 4C,D). We also investigated the effect of Cas9/gRNAs A and B on several parameters related to the health of the cells, including cell viability, cell cycle progression and apoptosis using several clonal cells after the eradication of HIV-1 by Cas9/gRNAs A and B. We found no persistent and significant deleterious effects on the host cells vital signs after elimination of the HIV-1 proviral DNA by the Cas9/gRNAs A and B (Figs S6–8).

To expand the scope of analysis of potential off-targets, we bioinformatically compared the InDel results from whole-genome sequencing of the 2D10 cells after treatment with the Cas9/gRNAs A/B system that elicited complete eradication of the proviral HIV-1 DNA. To improve InDel-calling confidence, we aimed the whole-genome sequencing at 100× coverage, but statistical analyses revealed that we actually achieved total coverage of 109.3× for control cells and 112.7× for HIV-1 eradicated cells (Table S1). Coverage levels varied for each chromosome, ranging >96× for chromosome 1 and >110× for chromosome 16 (Fig. S9). Using human (hg19) genome as a reference sequence, we identified 1,361,311 InDels (<50 bp insertions/deletions) in Control (+Cas9/−gRNA) and 1,358,399 in HIV-1-eradicated cells (Table S2), and 3,973,098 single nucleotide polymorphisms (SNP) in Control and 3,961,395 in HIV-1-eradicated cells (Table S3). Comparative bioinformatics analysis between the control and the HIV-1-eradicated cells identified 32,399 somatic InDels (small insertion/deletion called by Strelka), 46,614 somatic SNVs (single-nucleotide variations, called by MuTect) and 52 SVs (structural variations including large InDels called by CREST) between the latter two groups, that were distributed in different genomic regions (Table S4).

After discarding the small InDels we found in the public database dbSNP, we identified 30,156 InDels and 43,858 SNVs in HIV-1-eradicated cells. Filtering out heterozygous mutations, reduced this number to 989 InDels. To determine if these filtered InDels are *de novo* mutations caused by our Cas9/gRNA A/B editing system, we extracted ±30 bp, ±300 bp or ±600 bp sequences flanking each filtered InDel and used Blastn (e-value cutoff: 1000) to compare them *vs*. the potential gRNA off-target host genome sites predicted by sequence similarity at 0–7 mismatches, and *vs*. HIV-1 on-target sequences. Without any mismatches to targets of gRNAs A and B, we found no off-target site around the extracted 60, 600 and 1200 bp sequences of the filtered InDels. Within the extracted 60-bp sequences, we found no off-target site even with 7 mismatches at alignment lengths >12 nucleotides from PAM NRG (which must be 100% matched). Within the extracted 600-bp sequences, we found no off-target site with 3 mismatches for targets of gRNA A or B. With 4–7 mismatches, we found only one potential off-target site with 6 mismatches at an alignment length of 20 bp from PAM and another with 3 mismatches at 12 bp alignment length from PAM for Target A, and one additional potential off-target site with 4 mismatches at 16 bp length from PAM for Target B. Within the extracted 1200 bp sequences for 3 mismatches, we found no off-target sites for Target A but one potential off-target with 2 mismatches at 13 bp from PAM for Target B. With criteria of 3–7 mismatches against the 1200-bp sequences, we found only six potential off-target sites for Target A and two potential off-target sites for Target B (Fig. 4E). Together, these data strongly suggest that none of the indels detected in the cells with the excised HIV-1 genome lie within 60 bp of Targets A or B of any potential off-target sites, as predicted by search criteria allowing up to 7 mismatches. By expanding the searching sequences to 600 or 1200 bp, relatively rare off-target sites were identified, including various numbers of mismatches and aligned length. With perfect match to the last 12 bp seed sequence plus PAM NRG, none of the indels fell within the search area of 60–1200 DNA sequences. Our overall interpretation of these data verifies the preceding Surveyor assay results in these cells, as well as in the other cell types^25^, and establishes by very stringent analysis that no off-target effects upon the host T cell genome is elicited by our Cas9/gRNAs HIV-1 DNA-excising system.

### Infectivity of the HIV-1 eradicated cells by HIV-1

We selected several T-cell clones whose proviral DNA was eliminated by Cas9/gRNAs and maintained at various levels, expression of Cas9 as well as the gRNAs to assess the extent of new infection by HIV-1. As seen in Fig. S10A, clone C7 expresses Cas9 but not gRNA B, whereas clone AB8 shows no detectable level of Cas9, yet contains gRNA B. Two additional clones AB9 and AB5 with an equal amount of gRNA B and different levels of Cas9 expression were selected for a re-infection study. Infection of these cells by HIV-1_NL4-gfp_ followed by longitudinal evaluation of viral replication by flow cytometry showed that cells expressing either Cas9 or gRNA B alone were infectable by HIV-1, and supported viral replication throughout the course of these studies (day 18 post-infection) (Fig. S10B). In contrast, cells expressing both Cas9 and gRNA B were resistant to infection by HIV-1 and failed to support viral replication. AB5, which expressed a higher level of Cas9, appeared to be more resistant to viral replication than AB9, which showed reduced Cas9 expression (Fig. S10A,B). Figure S10C summarizes the quantitative values of the results shown in Panel B. The results demonstrate that the intracellular presence of both Cas9 and the LTR-directed gRNAs can effectively protect culture of human T-cells against new infection by HIV-1.

### Lentivirus mediated delivery of Cas9/gRNA suppresses HIV-1 infection of CD4+ T-cells

**Figure 5:**
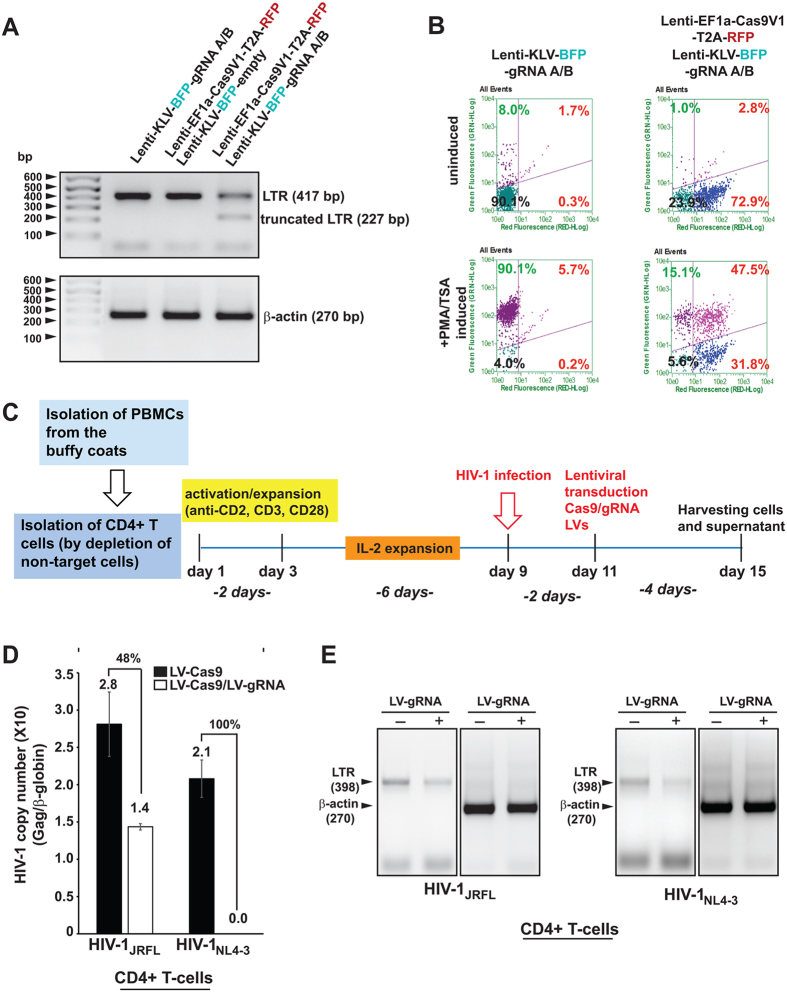
Lentivirus (LV) mediated Cas9/gRNA delivery suppresses HIV-1 infection in human T-cells. (**A**) PCR fragment analysis of 2D10 T-cells treated with LV expressing gRNAs A/B, Cas9, or both Cas9 and gRNAs A/B. The positions of the full-length amplicon (417 bp) and the smaller DNA fragment (227 bp) after excision of the 190 bp between gRNAs A and B are shown. Amplification of the 270 bp β-actin DNA fragment is shown as a control. (**B)** Representative scatter plots of GFP (HIV-1) and RFP (Cas9) expressing cells demonstrating that after LV infection 72.9% of 2D10 cells express Cas9, which after induction with PMA/TSA more than 45% of these cells (31.8%) show no evidence for GFP expression, indicative of HIV-1 DNA elimination. (**C)** Experimental procedure layout of *in vitro* infection experiments in primary CD4+ T cells. CD4+ T cells were isolated from freshly prepared, antibody labeled PBMCs by negative selection on magnetic columns (Miltenyi Biotec) and then activated with 48 h anti-CD2/CD3/CD28 treatment followed by 6 days human rIL-2 mediated expansion. Next cells were infected with HIV-1 by spinoculation and 2 days later transduced with lentiviral cocktails containing lenti-Cas9 with or without lenti-gRNA LTR A/B. 4 days later cells supernatants and cells were harvested and analyzed for HIV-1 presence. **(D)** CD4+ T-cells prepared from PBMC freshly isolated from buffy coat were infected with HIV-1_JRFL_ or HIV-1_NL4-3_ as described in Experimental Procedures, and HIV-1 copy number was determined by TaqMan qPCR and normalized to β-globin gene copy number. A significant reduction (48%) in the copy number of HIV-1_JRFL_ after 6 days of infection and even more dramatic decrease in HIV-1NL4-3 was observed upon LV-Cas9/gRNA expression in comparison to those that received LV-Cas9. **(E)** PCR analysis of the LTR and β-actin DNA (control) from the HIV-1 infected CD4+ T-cells treated with LV-Cas9 in the presence or absence of LV-gRNAs A/B. The positions of the 398 bp HIV-1 LTR and 270 bp β-actin amplicons are shown.

We tested the ability of Cas9/gRNAs to suppress HIV-1 infection of CD4+ T-cells prepared from healthy individuals. We chose a lentivirus vector for delivering Cas9 and gRNA expression DNAs because of its high transduction efficiency and low toxicity. Results of the LV transduction showed efficient cleavage of the HIV-1 LTR DNA by the LVs expressing both Cas9 and gRNAs, but not in control cells transduced with LV expressing only Cas9 (Fig. 5A). Of note, our gRNAs do not cleave the LVs LTR, which lacks the U3 modulatory region, thus they have no effect on the expression of the LV genome. Accordingly, flow cytometry analysis revealed functional inactivation of the integrated HIV-1 genome in latently-infected T-cells upon transduction with LV-Cas9/gRNA (Fig. 5B). Again, we found no evidence for cell death that may be associated with Cas9/gRNAs in the primary cells, corroborating our observations shown in Fig. S6. Once the efficacy of the gene delivery of editing molecule by LV was verified in the T-cell line, we infected primary cultures of CD4+ T-cells with HIV-1_JRFL_ or HIV-1_PNL4-3_, then transduced them with either control LV Cas9 or LV Cas9 plus LV gRNA (Fig. 5C). Compared to controls, a substantial decrease in HIV-1 copy number was seen in the CD4+ T-cells treated with LV Cas9/gRNA (Fig. 5D). Amplification of viral DNA revealed the expected 398-bp amplification in the control cells and a similar-sized DNA fragment with lesser intensity in cells transduced with LV Cas9/gRNA in CD4+ T-cells (Fig. 5E).

We also assessed the ability of lentivirus delivered Cas9/gRNA in editing the HIV-1 genome present in PBMC’s and CD4+ T-cells obtained from HIV-1+ patients during routine visits to the Temple University Hospital AIDS clinic. For this proof-of-concept study, initially we sought to prepare PBMCs and CD4+ T-cells from four patients (TUR0001 to TUR0004;Cases 1–4) who were undergoing antiretroviral therapy and exhibited diverse responses to treatment as determined by viral load assay and percentage of CD4+ cells (Fig. S11A). The procedure we used to prepare PBMCs and CD4+ T-cells and examine CD4+ T-cells, and the timeline for lentivirus treatments and cell harvest are shown in Fig. S11B. The purity of the CD4+ T-cells was confirmed by flow cytometry of FITC conjugated anti-CD4 antibody (Fig. S11C).

**Figure 6:**
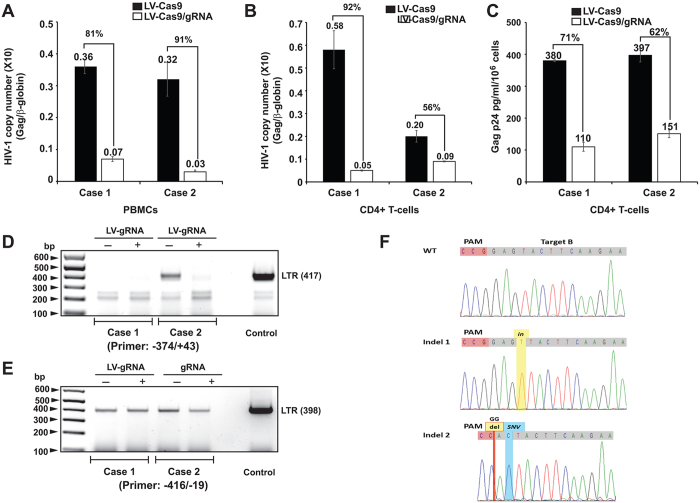
Suppression of HIV-1 replication in the peripheral blood mononuclear cells (PBMCs) and CD4+ T-cells of HIV-1 infected patients. (**A**) PBMCs from two HIV-1 infected volunteers were treated with LV-Cas9 or LV-Cas9 plus LV-gRNAs A/B (described in Materials and Methods) and viral DNA copies were determined by qPCR. As seen, a substantial decrease in the viral copy numbers was detected after normalization to β-globin DNA. (**B**) CD4+ T-cells isolated from PBMCs were expanded in media containing human IL-2 (20 U/ml) and infected with LV-Cas9 or LV-Cas9 plus LV-gRNA A/B followed by determination of viral DNA copy number 4 days later by qPCR. Similar to the PBMCs, a drastic reduction in the copy number of HIV-1 DNA was observed in cells receiving LV-Cas9/gRNAs compared to LV-Cas9. **(C)** CD4+ T-cells after treatment with lentivirus vector expressing Cas9 or Cas9 and gRNAs A/B were harvested and viral replication was determined by p24 Gag ELISA. **(D)** PCR analysis of DNAs isolated from the patient samples after lentivirus treatment by primers expanding −374/+43. The position of the 417 expected amplicons is shown. Control represent amplification of LTR DNA from PBMCs HIV-1_JRFL_ infected at 6 days of infection. **(E)** PCR amplification of viral LTR (as described in **D**) using a different set of primers spanning −416 – −19 of the LTR. The position of 398 bp amplicon is shown. **(F)** TA cloning and sequencing of the LTR fragment (shown in Panel **E**) from patient 2 showed insertion, deletion and single nucleotide variation (SNV) in some of the amplified DNA. Note that the assay cannot detect large DNA elimination that requires primers derived from the outside of the virus genome, i.e. flanking the site of integration.

Results of transducing PBMC’s with lentivirus-Cas9 and lentivirus-Cas9/gRNA revealed a substantial decrease, 81% in Case 1 and 91% in Case 2, in the viral copy number of cell populations expressing Cas9 and gRNA (Fig. 6A). Similar results were obtained after lentiviral transduction of CD4+ T-cells, which showed >92% reduction in viral copies in Case 1 and 56% for Case 2 upon expression of both Cas9 and gRNA, compared to control cells expressing only Cas9 (Fig. 6B). Standard curves and amplification plots served for absolute quantification of β-globin and Gag gene copy number is shown in Fig. S13. Examination of Gag p24 gene production in the CD4+ T-cells confirmed viral replication was decreased in Case 1 (71%) and Case 2 (62%) upon single transduction of the cells with lentivirus-Cas9/gRNA compared to that seen with lentivirus-Cas9 (Fig. 6C). Also, we examined the level of Gag p24 in PBMCs obtained from Cases 3 and 4 after delivery of Cas9/gRNA by lentivirus. Results from this study showed 39% and 54% decrease in HIV-1 p24 production from Cases 3 and 4, respectively, after transduction of the cells with therapeutic lentivirus (Fig. S12).

Next, we assessed the nature of mutations introduced by Cas9/gRNAs in the patient samples, by amplifying and sequencing of viral DNA. Our initial gene amplification of the CD4+ T-cells using primers spanning −374/+43 failed to detect any band in Case 1 and in Case 2 a DNA band was observed in the control sample that lacked gRNA expression (Fig. 6D). This observation suggests that the HIV-1 genome sequence in case 1 may differ from those of the primers we used for gene amplification (Fig. 6D). In case 2, where we detected the expected DNA fragment in untreated cells, the mutations that were introduced by Cas9/gRNA may have eliminated the recognition of DNA sequence by the PCR primer, thus interfering with DNA amplification. The use of an alternative set of primers that recognizes different regions of the LTR led to production of the expected 398-nucleotide amplicon in all samples (Fig. 6E). It is possible that similar to the results from 2D10 cells after treatment with Cas9/gRNAs (shown in Fig. 2A), some of the 397 nucleotide DNA fragments seen in the presence of gRNA expression result from joining of the remaining 5′ and 3′ sequences of the viral LTR after excision of the entire HIV-1 coding sequence. Sequencing of the amplicon verified the effect of Cas9/gRNA on editing of the viral genome at the expected positions and showed the presence of InDel and single nucleotide variation (SNV) mutations within and/or next to the PAM sequence within the LTR (Fig. 6F).

## Discussion

Despite its remarkable therapeutic success and efficacy, ART treatment is unable to eradicate HIV-1 from infected patients who must therefore undergo life-long treatment. A new therapeutic strategy is thus needed in order to achieve permanent remission allowing patients to stop ART and reduce it’s attendant costs and potential long-term side effects. Our findings address key barriers to this goal, as we developed CRISPR/Cas9 techniques that eradicated integrated copies of HIV-1 from human CD4+ T-cells, inhibited HIV-1 infection in primary cultured human CD4+ T-cells, and suppressed viral replication *ex vivo* in peripheral blood mononuclear cells (PBMCs) and CD4+ T-cells of HIV-1+ patients. They also address a further key issue, providing evidence that such gene editing effectively impedes viral replication without causing genotoxicity to host DNA or eliciting destructive effects via host cell pathways. Prior studies using gene editing based on zinc finger nuclease (ZFN), transcription activator-like effector nuclease (TALEN), and CRISPR/Cas9 systems prompted much interest in their potential abilities to suppress viral infection, either by altering virus receptors or introducing mutations in the viral genome (for review see^26^,^30^). All these studies suggest that gene editing strategies can be engineered for targeting specific regions of the viral genome and once efficiently delivered to infected cells, their robust antiviral activity effectively suppresses viral replication. However, there are several important issues that require close attention including the careful design of the targeting strategy that achieves the highest levels of specificity and safety with optimum efficiency of editing.

In this study, due to the complexity associated with determination of the sites and numbers of randomly integrated proviral HIV-1 DNA in *in vitro* infected primary cell culture and the difficulty in full scale characterization of the InDel/Excision by Cas9/gRNAs in these cells, as a first step, we chose to use the clonal 2D10 cell line as a human T-cell latency model to establish: (i) the ability of Cas9/gRNA in removing the entire coding sequence of the integrated copies of the HIV-1 DNA using ultradeep whole genome sequencing and (ii) investigate its safely related to off-target effects and cell viability. Once these goals were accomplished, we shifted our attention to primary cell culture as well as patient samples to examine the efficiency of the CRISPR/Cas9 in affecting viral DNA load in a laboratory setting.

We found that CRISPR/Cas9 edited multiple copies of viral DNA scattered among the chromosomes. Combined treatment of latently-infected T cells with Cas9 plus gRNAs A and B that recognize specific DNA motifs within the LTR U3 region efficiently eliminated the entire viral DNA fragment spanning between the two LTRs. The remaining 5′ LTR and 3′ LTR cleavage sites by Cas9 and gRNA B in chromosome 1, and by Cas9 and gRNAs A and B in chromosome 16, were joined by host DNA repair at sites located precisely three nucleotides upstream of the PAM. Genome-wide assessment of CRISPR/Cas9-treated HIV-1-infected 2D10 cells clearly verified complete excision of the integrated copies of viral DNA from the second intron of RSBN1 and exon 2 of MSRB1 genes. To address the critical issue related to its specificity and potential off-target and adverse effects, we analyzed this comprehensively and at an unprecedented level of detail, by whole-genome sequencing and bioinformatic analyses. These revealed many naturally-occurring mutations in the genomes of control cells and gRNAs A- and B-mediated HIV-1 DNA eradication. The mutations discovered included naturally-occurring InDels, base excisions, and base substitutions, all of which are, more or less, expected in rapidly growing cells in culture, including Jurkat 2D10 cells. The critical issue is our discovery that none of these mutations resulted from our gene-editing system, as we identified no sequence identities with either gRNA A or B within 1200 nucleotides of any such mutation site. Further, our method of HIV-1 DNA excision had no adverse effects on proximal or distal cellular genes and showed no impact on cell viability, cell cycle progression or proliferation, and did not induce apoptosis, thus strongly supporting its safety at this translational phase, by all *in vitro* measures assessed in cultured cells. We found that the expression levels of Cas9 and the gRNAs diminished after several passages and eventually disappeared, but as long as Cas9 and single or multiplex gRNAs were present, cells remained protected against new HIV-1infection.

Another key translational feasibility question we addressed is whether CRISPR/Cas9-mediated HIV-1 eradication can prevent or suppress HIV-1 infection in the most relevant human and patient target cell populations. We provide a critical new advance, by observing in PBMCs and CD4+ T-cells from HIV-1 infected patients that lentivirally-delivered Cas9/gRNAs A/B significantly decreased viral copy numbers and protein levels. Using primer sets directed within the LTR, we amplified and detected residual viral DNA fragments that were not completely deleted in these cells, yet were affected by Cas9/gRNAs and contained InDel mutants near the PAM sequence. These findings verified that CRISPR/Cas9 exerted efficacious antiviral activity in the PBMCs of HIV-1 patients. We also found that introducing Cas9/gRNAs A/B via lentiviral delivery into primary cultured human CD4+ HIV-1_JRFL_- or HIV-1_NL4-3_-infected T-cells significantly reduced viral copy numbers, corroborating earlier findings by us and others that stably-integrated HIV-1-directed Cas9 and gRNAs (distinct from our gRNAs A and B used presently) conferred resistance to HIV-1 infection in cell lines^31^,^32^. With the notion that CRISPR/Cas9 can target both integrated, as well as episomal DNA sequences, as evidenced by its editing ability of various human viruses as well as plasmid DNAs in either configuration^31^,^32^,^33^,^34^,^35^,^36^, it is likely that both the integrated as well as pre-integrated, free-floating intracellular HIV-1 DNA are edited by Cas9/gRNA.

As noted, during the course of our studies no ART was included prior to the treatment with CRISPR/Cas9 as our goal in this study was to determine the extent of viral suppression during the productive stage of viral infection. We observed a significant level of suppression suggesting that CRISPR/Cas9 may effectively disable expression of the functionally active integrated copies of HIV-1 DNA in the host chromosome. This notion is supported by our observations using 2D10 CD4+ T-cells where the latent copies of HIV-1 that are integrated in chromosomes 1 and 16 were effectively eliminated by CRISPR/Cas9. Our future studies are aimed to address the impact of CRISPR/Cas9 in *in vitro* infected CD4+ T-cells where the virus is controlled by ART and a cohort of naïve and ART-treated patient CD4+ T-cells. Results from these studies should determine whether or not, in the context of ART, the virus enters into the latent stage and remains responsive to CRISPR/Cas9. Of note, results from these *ex vivo* studies using ART treated patient PBMCs and CD4+ T-cells show that CRISPR/Cas9 effectively suppresses viral replication by introducing InDel mutations.

Our findings show comprehensively and conclusively that the entire coding sequence of host-integrated HIV-1 was eradicated in human 2D10 T cells, providing a strong first step of support for potential translatability of such a system to T-cell-directed HIV-1 therapies in patients. The complete absence of genomic and off-target functional effects in all assays also provides critical support for the promise of developing this approach for future therapeutic applications.

When evaluating a therapeutic strategy based on CRISPR/Cas9, it is critical to understand that not only will HIV-1 be eliminated from latently infected cells, but the majority of uninfected cells will become resistant to HIV infection. Thus, there is a high likelihood that rebounding viral infections will be contained by the resistant cells. Still, some formidable challenges remain before this type of strategy can be implemented. First, it will be important to maximize elimination of viral sequences from patients. This will require analysis of the HIV-1 quasi-species harbored by patients’ CD4+ T-cells and design of suitable, i.e. personalized CRISPRs. Second, improved delivery of CRISPR/Cas9 will be required to target the majority of circulating T-cells. In summary, our novel *ex vivo* findings that our lentiviral delivery-based approach reduced HIV-1 DNA copy numbers and protein levels in PBMCs of HIV-1 infected patients provides strong proof-of-concept evidence that CRISPR/Cas9 can be effectively utilized as part of HIV Cure strategies.

## Experimental Procedures

### Stable cell lines

Jurkat 2D10 reporter cell line has been described previously^11^ and cultured in RPMI medium containing 10% FBS and gentamicin (10 ug/ml). 2 × 10^6^ cells were electroporated with 10 ug control pX260 plasmid or pX260 LTR-A and pX260 LTR-B plasmids, 5 ug each (Neon System, Invitrogen, 3 times 10 ms/1350V impulse). 48 h later medium was replaced with one containing puromycin 0.5 ug/ml. After one week selection, puromycin was removed and cells were allowed to grow for another week. Next, cells were diluted to a concentration of 10 cells/ml and plated in 96 well plates, 50 ul/well. After two weeks, single cell clones were screened for GFP tagged HIV-1 reporter reactivation (12 h PMA 25 nM/TSA 250 nM treatment) using Guava EasyCyte Mini flow cytometer. The non-reactive clones were used for further analysis.

### Primary CD4+ cell isolation and expansion

Buffy coat was obtained through CNAC Basic Science Core I (Lewis Katz School of Medicine at Temple University, Philadelphia). PBMCs were isolated from human peripheral blood by density gradient centrifugation using Ficoll-Paque reagent. (For details, see S1 Experimental Procedures).

### Lentiviral delivery

Cloning lentiviral constructs as well as lentivirus packaging and purification, and transduction of primary culture are detailed in S1 Experimental Procedures.

### Virus stock, assays and detection

HIV-1_JRFL_ crude stock used was prepared from supernatants of PBMCs infected with HIV-1 for 6 days, clarified at 3000 RPM for 10 minutes and 0.45 um filtered. HIV-1_NL4-3_-EGFP-P2A-Nef reporter virus was prepared by transfecting HEK 293 T cells with pNL4-3-EGFP-P2A-Nef plasmid and processed like lentiviral stocks (see above). HIV-1_JRFL_ was titered using Gag p24 ELISA, HIV-1 NL4-3-EGFP-P2A-Nef by GFP-FACS of infected HEK 293 T cells.

*In vitro* HIV-1 infection, and viral detection and quantification including p24 ELISA, are described in S1 Experimental Procedures.

### Host genome analysis. Genomic DNA preparation, whole genome sequencing and bioinformatics analysis

The single subclone control C11 and experimental AB5 from parent 2D10 T cells were validated for target cut efficiency and functional suppression of HIV-1 EGFP reporter reactivation. The genomic DNA was isolated with NucleoSpin Tissue kit (Macherey-Nagel) according to the protocol of the manufacturer. The genomic DNA was submitted to Novogene Bioinformatics Institute (http://www.novogene.com/en/) for WGS and bioinformatics analysis. Briefly, DNA quality was further verified on 1% agarose gels, DNA purity was checked using the NanoPhotometer® spectrophotometer (IMPLEN, CA, USA) and DNA concentration was measured using Qubit® DNA Assay Kit in Qubit® 2.0 Fluorometer (Life Technologies, CA, USA). The detailed procedures for genomic DNA preparation, sequencing and bioinformatics, and SURVEYOR assays are described in S1 Experimental Procedures.

### Reverse transcription and PCR

Total RNA was extracted from Jurkat cells using RNeasy kit (Qiagen) with on column DNAse I digestion. Next 0.5 ug of RNA was used for M-MLV reverse transcription reactions (Invitrogen). For gRNA expression screening specific reverse primer (pX260-crRNA-3′/R, Table I.3 in S1 Experimental Procedures) was used in RT reaction followed by standard PCR using target A or B sense oligos as a forward primers (Table I.5 in S1 Experimental Procedures) and agarose gel electrophoresis. For checking neighboring genes expression oligo-dT primer mix was used in RT and cDNA was subjected to SybrGreen real time PCR (Roche) using mRNA specific primer pairs and β-actin as a reference (Table I in S1 Experimental Procedures).

### Flow cytometry

GFP and RFP expression in Jurkat 2D10 cells was quantified in live cells using Guava EasyCyte Mini flow cytometer (Guava Technologies). For HIV-1 reporter virus titer, cells were trypsinized 48 h after infections, washed and fixed in 4% paraformaldehyde for 10 minutes, then washed 3 times in PBS and analyzed for GFP FACS. CD4 expression in primary T cells was checked by direct labeling with CD4 V5 FITC antibody (BD Biosciences) followed by FACS.

### Anexin assay

Jurkat cells were washed, counted and diluted to a density of 1 × 10^5 ^cells/ml in PBS. For each sample, 100 uL of cells in suspension was mixed with 100 ul of room-temperature annexin V-PE staining reagent (Guava Nexin Reagent) and incubated for 20 minutes at room temperature in the dark. After incubation, samples were acquired using a Guava EasyCyte Mini flow cytometer.

Cell viability was assessed using propidium iodide staining. To 200 ul of live cells in suspension PI solution was added to final concentration 10 ug/ml. Samples were incubated for 5 minutes at room temperature in the dark. After incubation, samples were acquired using a Guava EasyCyte Mini flow cytometer.

### Cell cycle analysis

Cells were washed with 1× PBS and then resuspended in 250 ul of 1× PBS at room temperature. This suspension was added drop-wise to 1ml of −20 °C 88% ethanol, for a final concentration of 70% ethanol. Cells were fixed overnight at −20 °C then washed, incubated with 10 ug/ml of propidium iodide and RNase A solution 100 ug/ml in 1× PBS for 30 minutes at 37 °C then samples were cooled to 4 °C and acquired using a Guava EasyCyte Mini flow cytometer.

### Western-blot, immunocytochemistry

Standard methods for protein expression including Western blot and immunocytochemistry were used. For details see S1 Experimental Procedures.

## Ethics Statement

For *in vitro* HIV-1 infection studies, Buffy coat was obtained from healthy individuals by purchasing from Biological Specialty Corporation (Colmar, PA).

## Additional Information

**How to cite this article**: Kaminski, R. *et al.* Elimination of HIV-1 Genomes from Human T-lymphoid Cells by CRISPR/Cas9 Gene Editing. *Sci. Rep.* **6**, 22555; doi: 10.1038/srep22555 (2016).

## Supplementary Material

Supplementary Information
