# Corrigendum: Elimination of HIV-1 Genomes from Human T-lymphoid Cells by CRISPR/Cas9 Gene Editing

**DOI:** 10.1038/srep28213

**Published:** 2016-07-07

**Authors:** Rafal Kaminski, Yilan Chen, Tracy Fischer, Ellen Tedaldi, Alessandro Napoli, Yonggang Zhang, Jonathan Karn, Wenhui Hu, Kamel Khalili

Scientific Reports 6: Article number: 2255510.1038/srep22555; published online: 03042016; updated: 07072016

The Competing financial interests statement in this Article should read:

“KK, WH, RK, and YZ are named inventors on patents that cover the viral gene editing technology that is the subject of this published journal article. In addition to the foregoing interests, KK is a co-founder, board member, scientific advisor, and holds equity in Excision Biotherapeutics, a biotech start-up which has licensed the viral gene editing technology from Temple University for commercial development and clinical trials. The option to license the viral gene editing technology was signed prior to the acceptance of this manuscript. The authors declare that this work was produced solely by the authors and that no other individuals or entities influenced any aspects of the work including, but not limited to, the study conception and design; data acquisition, analysis and interpretation; and writing of the manuscript. The work was funded by the National Institutes of Health. No other entities provided funds for the work. The authors further declare that they have received no financial compensation from any other third parties for any aspects of the published work”.

