# Supplementary material for: Elimination of HIV-1 Genomes from Human T-lymphoid Cells by CRISPR/Cas9 Gene Editing
Source: Sci Rep. 2016 Mar 4;6:22555. doi: 10.1038/srep22555 (PMC4778041; doi:10.1038/srep22555)
Supplement: Supplementary Information [file srep22555-s1.pdf]

1/7/2016

Elimination of HIV-1 Genomes from Human T-lymphoid Cells by CRISPR/Cas9 Gene Editing

SUPPLEMENTARY MATERIALS

Rafal Kaminski[1,2†], Yilan Chen[1,2], Tracy Fischer[1,2], Ellen Tedaldi[2,3], Alessandro Napoli[1,2], Yonggang Zhang[1,2], Jonathan Karn[4], Wenhui Hu[1,2†], Kamel Khalili[1,2†*]

Affiliations:

1. Department of Neuroscience/Center for Neurovirology, Temple University School of Medicine, 3500 N. Broad Street, 7th Floor, Philadelphia, PA 19140

2. Comprehensive NeuroAIDS Center, Temple University School of Medicine, 3500 N. Broad Street, 7th Floor, Philadelphia, PA 19140

3. Department of Medicine, Temple HIV Program, Temple University School of Medicine 3400 N. Broad Street, Philadelphia, PA 19140

4. Department of Molecular Biology and Microbiology, Case Western Reserve University, Cleveland, OH 44106, USA.

†Corresponding authors. kamel.khalili@temple.edu (*senior corresponding author); wenhui.hu@temple.edu; rafalkim@temple.edu

S1. SUPPLEMENTARY EXPERIMENTAL PROCEDURES

Primary CD4+ cell isolation and expansion.

Blood/buffy coat samples volume was adjusted to 30 ml with HBSS buffer, gently layered on 15 ml of Ficoll-Paque cushion and centrifuged for 30 minutes at 1500 RPM. The PBMC layer was collected, washed 3 times in HBSS buffer and counted. Further isolation of CD4+ T-cells was performed using CD4+ T cell isolation kit (human) (Miltenyi Biotec). Cells (10^7) were labeled with biotin-conjugated antibody cocktail (anti-CD8, CD14, CD15, CD16, CD19, CD36, CD56 ,CD123, CD235a, TCRɣ/δ), then mixed with MicroBeads conjugated with anti-biotin and anti-CD61 antibodies and separated on MACS LS columns. Flow-through unlabeled cells representing the CD4+ enriched fraction were collected and purity was confirmed by CD4-FITC FACS (94-97% CD4+ positive, see Fig. S12). Next, cells were expanded using T-cell activation/expansion kit according to the manufacturer's protocol (Miltenyi Biotec). Briefly, 2.5 x 10^6 cells/ml were mixed with anti-CD2, CD3, CD28 antibodies covered MicroBeads in ratio of cells:beads of 2:1. After 2 days, cells were gently pipetted to disrupt clamps and one volume of fresh growth medium containing human rIL-2 was added. Medium was replaced every 3 days. All primary cells were grown in RPMI with 10% FBS and gentamicin (10 ug/ml) supplemented with human rIL-2 at concentration of 20 U/ml (NIH AIDS Reagent Program, Division of AIDS, NIAID, NIH: Human rIL-2 from Dr. Maurice Gately (Hoffmann-La Roche Inc.,). All procedures involving AIDS patient samples and in vitro infected cells were performed in BL2+ lab.

Lentiviral delivery

1. Cloning lentiviral constructs. The “all-in-one” pX260-U6-DR-BB-DR-Cbh-NLS-hSpCas9-NLS-H1-shorttracr-PGK-puro (Addgene 42229) vectors containing LTR target A and B were described previously (1). For lentiviral delivery into primary cells, DNA segments expressing gRNA for LTR target A and B were shortened to 20 nucleotides (Table I 5.) and first subcloned into U6-chimeric-gRNA expressing cassette of pX330-U6-Chimeric_BB-CBh-hSpCas9 (Addgene 42230) then

whole gRNA expressing cassette was PCR amplified with Mlu1/BamH1 extended primers (T560/T561 see Table I 5.), digested and inserted into Mlu1/BamH1 sites of pKLV-U6gRNA(Bbs1)-PGKpuro2ABFP (Addgene 50946).

2. Lentivirus packaging and purification. The obtained pKLV-U6-LTR A/B-PGKpuro2ABFP were packaged into lentiviral particles by co-transfection of HEK293T cells with pMDLg/pRRE (Addgene 12251), pRSV-Rev (Addgene 12253) and pCMV-VSV-G (Addgene 8454). For packaging Cas9 into lentiviral particles following vectors were used: pCW-Cas9 (Addgene 50661), psPAX2 (Addgene 12260) and pCMV-VSV-G (Addgene 8454). For some experiments pLV-EF1a-Cas9v1-T2A-RFP lentivirus was used (Biosettia Inc.) HEK 293T cells were co-transfected using $CaPO_4$ precipitation method in the presence of chloroquine (50 uM) with packaging lentiviral vectors mixtures at 30 ug total DNA/2.5 x 10^6 cells/100 mm dish. The next day, the medium was replaced and at 24 and 48h later supernatants were collected, clarified at 3000 RPM for 10 minutes, 0.45 um filtered and concentrated by ultracentrifugation (2h, 25000 RPMI, with 20% sucrose cushion). Lentiviral pellets were resuspended in HBSS by gentle agitation overnight, aliquoted and tittered in HEK 293T cells. pCW-Cas9 lentivirus was tittered by FLAG immunocytochemistry, pKLV-U6-LTR A/B-PGKpuro2ABFP lentiviruses by BFP fluorescent microscopy.

3. Lentiviral transduction of primary cells. 24h before transduction, growth medium was replaced and cells were activated by incubation with anti-CD2/CD3/CD28 antibody coated magnetic beads (Miltenyi Biotec) at cells/beads ratio 2:1. Next day 2.5x10^5 cells were infected with 12.5 x 10^5 IU of pCW-Cas9 lentivirus together with 25x10^5 IU pKLV-empty lentivirus or 12.5x10^5 IU of each pKLV-LTR target A and pKLV-LTR target B lentiviruses (total MOI 15). Cells were spinoculated for 2h at 2700 RPM, 32 °C in 150 ul inoculum containing 8 ug/ml polybrene then resuspended

and left for 4h, then 150 ul of growth medium was added. Next day cells were washed 3 times in 1 ml of PBS and incubated in growth medium containing human rIL-2 (20 U/ml).

Virus infection, assays and detection

1. In vitro HIV-1 infection. CD4+ T-cells prepared from primary PBMCs were activated and expanded for one week before HIV-1 infection. Infection was done using crude HIV-1 stocks at 300 ng of Gag p24/10^6 cells/1 ml by spinoculation for 2h at 2700 RPM, 32 °C in serum free medium containing 8ug/ml polybrene then resuspended and left for 4h followed by washing 3 times in PBS and finally incubated in growth medium containing human rIL-2 (20 U/ml). In case of CD4+ T cells infection, cells were activated and expanded for one week before HIV-1 infection. Jurkat 2D10 cells were reinfected without spinoculation by simple overnight incubation of the cells with diluted viral stock in the presence of polybrene (8 ug/ml).

2. HIV-1 DNA detection and quantification. Genomic DNA was isolated from cells using NucleoSpin Tissue kit (Macherey-Nagel) according to the manufacturer's protocol. For LTR specific PCRs (see Table I 1.), 100 ng of extracted DNA was subjected to PCR using Fail Safe PCR kit and buffer D (Epicentre) under the following conditions: 98 °C, 5 minutes, 30 cycles (98 °C 30 s, 55 °C 30 s, 72°C 30 s), 72 °C 7 minutes and resolved in 2% agarose gel. Integration site specific PCRs (see Table I 2.) were performed on 250 ng of genomic DNA using Long Range PCR kit (Qiagen) under following conditions: 93 °C 3 minutes, 35 cycles (93 °C 15 s, 55 °C 30 s, 62 °C 7.5 minutes). PCR products were subjected to agarose gel electrophoresis, gel purified, cloned into TA vector (Invitrogen) and sent for Sanger sequencing (Genewiz). HIV-1 DNA was quantified using TaqMan qPCR specific for HIV-1 Gag gene and cellular beta-globin gene as a reference (see Table I 6.). Prior to qPCR, genomic DNA from infected cells was diluted to 10 ng/ul and then 5 ul (=50 ng) was taken per reaction/well. Reaction mixtures were prepared using Platinum Taq DNA Polymerase (Invitrogen) according simplified procedure as described

previously. Standard was prepared from serial dilutions of U1 cells (NIH AIDS Reagent Program, Division of AIDS, NIAID, NIH: HIV-1 infected Cells (U1) from Dr. Thomas Folks (2) genomic DNA since it contains two single copies of HIV-1 provirus per diploid genome equal to beta-globin gene copy number. qPCR conditions for Gag gene: 98 °C 5 minutes, 45 cycles (98 °C 15 s, 62 °C 30 s with acquisition) for beta-globin gene: 98 °C 5 minutes, 45 cycles (98 °C 15 s, 62 °C 30 s with acquisition, 72 °C 1 minute). Reactions were carried out and data analyzed in a LightCycler480 (Roche).

Host genome analysis (continued from main text)

Genomic DNA preparation, whole genome sequencing and bioinformatics analysis. A total amount of 1.5 µg DNA per sample was used for sequencing library generation using Truseq Nano DNA HT Sample preparation Kit (Illumina USA) following manufacturer's recommendations and index codes were added to attribute sequences to each sample. The DNA sample was fragmented by sonication to a size of 350 bp, then DNA fragments were end-polished, A-tailed, and ligated with the full-length adapter for Illumina sequencing with further PCR amplification. At last, PCR products were purified (AMPure XP system) and libraries were analyzed for size distribution by Agilent2100 Bioanalyzer and quantified using real-time PCR. The clustering of the index-coded samples was performed on a cBot Cluster Generation System using Hiseq X HD PE Cluster Kit (Illumina) according to the manufacturer's instructions. After cluster generation, the library preparations were sequenced on an Illumina Hiseq X Ten platform and paired-end reads were generated.

The original raw data was transformed to sequenced reads by base calling and recorded in FASTQ file, which contains sequence information (reads) and corresponding sequencing quality information. After filtering out any reads with adapter (>10 nucleotide aligned to the adaptor, allowing ≤10% mismatches), ≥10% unidentified nucleotides, >50% bases having phred quality, a total of 342.67 Gb clean reads (average 109.25x coverage) for the control sample and

369.55 Gb (112.72x) for AB5 sample were retained for further assembly. Burrows-Wheeler Aligner (BWA) software (3) was utilized to map the paired-end clean reads to the reference human genome (UCSC hg19) and HIV-1 genome (KM390026.1). Then, Picard Samtools (4) (http://broadinstitute.github.io/picard/), and GATK (5) were used to do duplicate marking, local realignment, and base quality recalibration to generate final BAM file for computation of the sequence coverage and depth. Candidate indels were filtered on several criteria using Python and the PyVCF (version 0.6.0), and PyFasta packages (version 0.5.0). We focused on the potential off-target effect of Cas9/LTR-gRNAs (AB5 group) on host genome by comparing the difference between the control (C11) and the experimental group (AB5). The SNV was detected by muTect (6), the indel by Strelka (7) and the structural variants (SV) by CREST (8). The total number of indels unique in AB5 group was 32,399, and filtered by public database (dbSNP) (9) and heterozygous indels. Then, we extracted sequences from 300 bp (600bp) upstream to 300 bp (600 bp) downstream of the indel sites as described previously (1, 10). and compared them to the predicted potential off-target sequence LTR-A/B + NRG. Similarly, SV analysis detected 42 deletions and 10 insertions in AB5 group, and the extraction sequences at ± 300 bp (600 bp) were compared against predicted off-target sequence LTR-A/B + NRG. To determine the integration site(s) of HIV-1, we used CREST (11) to detect the SV of the control sample that related to HIV-1 genome.

Western blot, immunocytochemistry (continued from main text)

Whole cell lysates were prepared by incubation of Jurkat cells in TNN buffer (50 mM Tris pH 7.4, 150 mM NaCl, 1% Nonidet P-40, 5 mM EDTA pH 8, 1x protease inhibitor cocktail for mammalian cells (Sigma) for 30 minutes on ice then precleared by spinning at top speed for 10 minutes at 4 °C. 50 ug of lysates were denatured in 1x Laemmli buffer and separated by SDS-polyacrylamide gel electrophoresis in Tris-glycine buffer followed by transfer onto nitrocellulose membrane (BioRad). The membrane were blocked in 5% milk/PBST for 1h and then incubated with mouse

anti-flag M2 monoclonal antibody (1:1000, Sigma) or mouse anti-α-tubulin monoclonal antibody (1:2000). After washing with PBST, the membranes were incubated with conjugated goat anti-mouse antibody (1:10,000) for 1h at room temperature. The membranes were scanned and analyzed using an Odyssey Infrared Imaging System (LI-COR Biosciences).

Cells were cultured in 4-well chamber slides and next day fixed with 4% paraformaldehyde/PBS for 10 min. After 3 times washing, cells were incubated in 0.1% Triton X-100, 2% BSA/ PBS with mouse anti-flag M2 monoclonal antibody (1:1000, Sigma) at room temperature for 2h. After washing 3 times, cells were incubated with goat anti-mouse FITC secondary antibody (1:200), and then incubated with Hoechst 33258 for 5min. After 3 rinses with PBS, the cells were coverslipped with anti-fading aqueous mounting media (Biomeda) and analyzed under a Leica DMI6000B fluorescence microscope.

Table I: Sequences of DNA oligonucleotides used in this study

primer	sequence
**1. PCRs**	
LTR -453/S	5'-TGGAAGGGCTAATTCACTCCCAAC-3'
LTR -374/S	5'-TTAGCAGAACTACACACCAGGGCC-3'
LTR +43/AS	5'-CCGAGAGCTCCCAGGCTCAGATCT-3'
LTR -417/S	5'-GATCTGTGGATCTACCACACACA-3'
LTR -19/AS	5'-GCTGCTTATATGTAGCATCTGAG-3'
RRE/S	5'-CGCCAAGCTTGAATAGGAGCTTTGTTCC-3'
RRE/AS	5'-CTAGGATCCAGGAGCTGTTGATCCTTTAGG-3'
LTR-A-OT-1/S	5'-GTGGACTTTGGATGGTGAGATAG-3'
LTR-A-OT-1/AS	5'-GCCTGGCAAGAGTGAACTGAGTC-3'
LTR-A-OT-2/S	5'-AAGATAATGAGTTGTGGCAGAGC-3'
LTR-A-OT-2/AS	5'-TCTACCTGGTAATCCAGCATCTGG-3'
LTR-A-OT-3/S	5'-ATAGGAGGAAGGCACCAAGAGGG-3'
LTR-A-OT-3/AS	5'-AATGATGCTTTGGTCCTACTCCT-3'
LTR-A-OT-4/S	5'-TGCTCTTGCTACTCTGGCATGTAC3'
LTR-A-OT-4/AS	5'-AATCTACCTCTGAGAGCTGCAGG-3'
LTR-A-OT-5/S	5'-TCAGACACAGCTGAAGCAGAGGC-3'
LTR-A-OT-5/AS	5'-ATGCCAGTGTCAGTAGATGTCAG-3'
LTR-A-OT-6/S	5'-TCAAGATCAGCCAGAGTGCACATG-3'
LTR-A-OT-6/AS	5'-TGCTCTTCCGAGCCTCTCTGGAG-3'
b- actin S	5'-CTACAATGAGCTGCGTGTGGC-3'
b-actin AS	5'-CAGGTCCAGACGCAGGATGGC-3'
**2. Long range PCR**	
D10-Chr1-5'Arm/F(6-29)	5'-GAGCACAGGACTCATTCAACAGT-3'
D10-Chr1-3'Arm/R(276-250)	5'-TTTGTATGTCAACAGACAGTATCCAG-3'
D10-Ch16 MSRB1-S	5'-TGTGCATACTTCGAGCGGCT-3'
D10-Ch16 MSRB1-AS	5'-GGAAAGGCGGGAGCTGATGA-3'
**3. gRNA RT and PCR**	
pX260-crRNA-3'/R	5'-TGGGACCATTCAAAACAGCAT-3'
**4. Neighboring genes qPCR**	
RSBN1/F	5'-GTAAGGCCAGGAGAACAGATG-3'
RSBN1/R	5'-TCAAAGAGAACTTCGCGGG-3'
PHTF1/F	5'-CCCAAGTTGTGTCCATCCTATC-3'
PHTF1/R	5'-AGACACCCCATTACCCAAAC-3'
MAGI3/F	5'-GACACCGCAGTAATTTCAGTTG-3'
MAGI3/R	5'-AGCAAGACGAAGGATGAACAG-3'
PTPN22/F	5'-TTTGCCCTATGATTATAGCCGG-3'
PTPN22/R	5'-GTTGTAGATAAAGGACCCTGGG-3'
AP4B1-AS1/F	5'-AGAAGGAAAAGGAGCAGACAC-3'
AP4B1-AS1/R	5'-AGAAAGTGGAGGTGCTGTG-3'
HS3ST6/F	5'-CTTCTACTTCAACGCCACCA-3'
HS3ST6/R	5'-AAGGGCCGGTAGAACTCC-3'
RPL3L/F	5'-AACAATGCATCCACCAGCTA-3'
RPL3L/R	5'-GTAATGACCCGCTTCTTGGT-3'
MSRB1/F	5'-GAAGCTTAGGCCCACATCTC-3'
MSRB1/R	5'-CTGGAAGGGTTTGACCAGAG-3'
NDUFB10/F	5'-GCATGTATGAAGCCGAAATG-3'
NDUFB10/R	5'-TGAACTGCTCCACTTCCTTG-3'
RPS2/F	5'-GCCTCTCTCAAGGATGAGGT-3'
RPS2/R	5'-CAACAAATGCCTTGAACCTG-3'
b- actin S	5'-CTACAATGAGCTGCGTGTGGC-3'
b-actin AS	5'-CAGGTCCAGACGCAGGATGGC-3'

**5. Target A and B oligos and cloning**	
LTR-A S/F/5’	5’-CACCGATCAGATATCCACTGACCTT-3’
LTR-A S/R/3’	5’-AAACAAGGTCAGTGGATATCTGATC-3’
LTR-B AS/F/5’	5’-CACCGCAGCAGTTCTTGAAGTACTC-3’
LTR-B AS/R/3’	5’-AAACGAGTACTTCAAGAACTGCTGC-3’
T560	5’-TATGGGCCCACGCGTGAGGGCCTATTTCCCATGAT TCC-3’
T561	5’-TGTGGATCCTCGAGGCGGGCCATTTACCGTAAGTT ATG-3’
**6. Taqman qPCR**	
HIV-Gag-RTfw	5’-CATGTTTTCAGCATTATCAGAAGGA-3’
HIV-Gag-RTrev	5’-TGCTTGATGTCCCCCCACT-3’
HIV-RTprobe	5'-/56-FAM/-CCACCCCACAAGATTTAAACACC-BHQ-3'
b-globinRTfw	5'-CCCTTGGACCCAGAGGTTCT-3'
b-globinRTrev	5'-CGAGCACTTTCTTGCCATGA-3'
b-globinRT probe	5'-FAM-GCGAGCATCTGTCCACTCCTGATGCTGTTATGG GCGCTCGC-TAMRA-3'

Statistical Analysis

The represented ±SD were from three experiments and were evaluated by student t-test or ANOVA and Newman-Keals multiple comparison test. In general, a p value < 0.05 or 0.01 was considered as statisically significant.

References

1. Hu W, et al. RNA-directed gene editing specifically eradicates latent and prevents new HIV-1 infection. *Proc Natl Acad Sci USA* **111**, 11461-11466 (2014).

2. Folks TM, Justement J, Kinter A, Dinarello CA, Fauci AS. Cytokine-induced expression of HIV-1 in a chronically infected promonocyte cell line. *Science* **238**, 800-802 (1987).

3. Li H, Durbin R. Fast and accurate short read alignment with Burrows-Wheeler transfrom. *Bioinformatics* **25**, 1754-1760 (2009).

4. Li H, et al. 1000 Genome Project Data Processing Subgroup. The sequence alignment/map format and SAMtools. *Bioinformatics* **25**, 2078-2079 (2009).

5. DePristo MA, et al. A framework for variation discovery and genotyping using next

generation DNA sequencing data. *Nat Genetics* **43**, 491-498 (2011).

6. Cibulskis K, et al. Sensitive detection of somatic point mutations in impure and heterogenous cencer samples. *Nat Biotechnol* **31**, 213-219 (2013).

7. Saunders CT, et al. Strelka: accurate somatic small variant calling from sequenced tumor-normal sample pairs. *Bioinformatics* **28**,1811-181 (2012).

8. Wang J, et al. CREST maps somatic strcutural variation in cancer geomes with base-pair resolution. *Nat Methods* **8**, 652-654 (2011).

9. Sherry ST, et al. dbSNP: the NCBI database of genetic variation. *Nucl Acids Res* **29**, 308-311 (2001).

10. Veres A, et al. Low incidence of off-target mutations in individual CRISPR/Cas9 and TALEN targeted human stem cell clones detected by whole genome sequencing. *Cell Stem Cell* **15**, 27-30 (2014).

11. Wang J, Quake SR. RNA-guided endonuclease provides a therapeutic strategy to cure latent herpesviridae infection. *Proc Natl Acad Sci USA* , 13157-13162 (2014).

Supplementary Figure Legends

Figure S2. A. Flow cytometry evaluation of several 2D10 clones transfected with plasmids expressing either Cas9 or Cas9 plus gRNAs. Treatment of the +Cas9/-gRNAs cells with PMA/TSA induced HIV-1 expression (GFP+) in 71% - 89% of the cells. Conversely, cells transfected with Cas9 and gRNAs showed no significant response (1% - 3%) to the treatment. **B.** RT-PCR assay for detection of gRNAs A and B in several clonal 2D10 cells after eradication of their latent HIV-1 genome. β-actin mRNA levels served as a control for the integrity of RNA preparation and loading. C11 represents RT-PCR of control (+Cas9/-gRNA) cells.

Figure S3. Whole-genome sequencing and bioinformatic analysis of human T cells harboring integrated copies of HIV-1 proviral DNA. A. Details of the HIV-1 integration sites at the nucleotide levels on Chromosomes 1 and 16 are shown on the right. The host chromosomal DNA sequences are shown in *red* and the integrated DNA sequences are shown in *black*. Four deleted nucleotides (TAAG) are underlined in *green*. Four inter-chromosomal translocations (CTX) associated with HIV-1 are identified based on CREST calling of structural variants. **B.** Graphic representation of chromosomes 1 and 16, analyzed by NCB1, BLASTIN, highlights the correspondence between the HIV-1 genome and host chromosomes. (LTR, long terminal repeats)

Figure S4. Results from DNA sequencing of the portion of Chromosome 1 depicting regions within RSNB1 where HIV-1 DNA is integrated. A. The positions of PAM along with nucleotide sequences of the LTR corresponding to gRNAs A and B (LTR A and B) are highlighted. **B.** DNA sequencing of PCR fragment showing the precise position of breakpoint and the seven nucleotide insertion at 3 nucleotides downstream from PAM.

Figure S5. A. DNA sequencing of host DNA in Chromosome 16 illustrating the precise sites of HIV-1 DNA integration within the MRSB1 gene and highlighted areas of InDel mutation. **B.** The

positions of insertion of 8 nucleotides within the 5'-LTR after cleavage by gRNA A (at LTR A target) and insertion of 3 nucleotides upon the cleavage by gRNA B (at LTR B target) are shown.

Figure S6. Apoptotic assay was used to assess the impact of Cas9/gRNA for eradication of HIV-1 on cell apoptosis. The top bar graph shows the average results of the apoptotic assay performed on 14 T-cell clones infected only with Cas9 lentivirus and no gRNAs. For each sample the experiments were performed in triplicate, data are presented as average and standard deviation. The different colors represent the average percentage of cells detected in the different apoptotic stages, as shown in the table underneath the graph. The top-left panel shows the results for a representative sample. The bottom bar graph shows the results of the same apoptotic assay carried out on T-cell clones in which HIV-1 had been previously eradicated by infecting the cells with both Cas9 and gRNA lentiviruses. The bottom-left panel shows the results for a representative sample. The results show no significant differences between clones infected with Cas9 and eradicated ones, showing that gRNAs do not affect apoptotic cellular mechanisms.

Figure S7. Cell viability assay was used to investigate the impact of Cas9/gRNAs developed for HIV-1 eradication on cell viability. The top bar graph shows the average results of the cell viability assay performed on 14 T-cell clones infected only with Cas9 lentivirus and no gRNAs. For each sample, the experiments were performed in triplicate, data are presented as average and standard deviation. The average percentage of live and dead cells is displayed respectively with blue and red. The top-left panel shows the results for a representative sample. The bottom bar graph shows the results of the same cell viability assay carried out on T-cell clones in which HIV-1 had been previously eradicated by infecting the cells with both Cas9 and gRNA lentiviruses. The bottom-left panel shows the results for a representative sample. The results show no significant differences between clones infected with Cas9 and eradicated ones, showing that gRNA lentiviruses do not induce cell death.

Figure S8. Cell cycle asssay was used to investigate the impact of Cas9/gRNAs developed for eradication of HIV-1 on cell cycle. The top bar graph shows the average results of the cell cycle assay performed on 14 T-cell clones infected only with Cas9 lentivirus and no gRNAs. For each sample the experiments were performed in triplicate, data are presented as average and standard deviation. The average percentage of cells detected in the different cell cycle phases are displayed in different colors, as shown in the table underneath the bar graph. The top-left panel shows the results for a representative sample. The bottom bar graph shows the results of the same cell cycle assay carried out on T-cell clones in which HIV-1 had been previously eradicated by infecting the cells with both Cas9 and gRNA lentiviruses. The bottom-left panel shows the results for a representative sample. The results show no significant differences between clones infected with Cas9 and eradicated ones, showing that gRNA lentiviruses do not affect cell cycle mechanisms.

Figure S9. The coverage depth (the left coordinate) and coverage rate (the right coordinate) of chromosome. The X-axis is chromosome number, the left Y-axis is the average depth of each chromosome, the right Y-axis is the fraction covered on each chromosome.

Figure S10. Protection of HIV-1 excised T-cell line from re-infection. A. Several latently infected T-cells after elimination of their HIV-1 genome were examined for expression of Cas9 (top panel) by Western blot and the presence of gRNA B (middle panel) and by RT-PCR. Expression of α-tubulin and β-actin serve as the loading controls for protein and RNA, respectively. **B.** T-cells with expression of Cas9 and/or gRNAs were infected with HIV-1 and at various times post-infection, the level of viral infection in each case was determined by flow cytometry. **C.** Quantitative values of the experiment shown in Panel B.

Figure S11. Patient derived primary PBMCs and CD4+ T-cell experiments. A. Blood samples from four HIV-1 positive patients on ART were obtained through the CNAC Basic Science Core 1 (Temple University, Philadelphia). AA: African-American, His: Hispanic. B. Schematic representation of experimental workflow for patient blood samples. CD4+ T-cells were isolated from freshly prepared, antibody labeled PBMCs by negative selection on magnetic columns (Miltenyi Biotec) and then activated with 48 hours anti-CD2/CD3/CD28 treatment followed by 6 days human rIL-2 mediated expansion. In parallel, PBMCs from the same blood samples were PHA-activated and similarly expanded with human rIL-2. Next, cells were transduced with lentiviral cocktails containing lenti-Cas9 with or without lenti-gRNA LTR A/B. 4 days later, supernatants and cells were harvested and analyzed for HIV-1 presence. C. The purity of CD4+ T-cells after isolation was checked by flow cytometry of FITC-conjugated anti-CD4 antibody labeled cells. Representative histograms of CD4 positive (GFP channel) cells after isolation in CD4 depleted and enriched populations.

Figure S12. HIV-1 levels in patient derived PBMCs. p24 ELISA assay of PBMCs from Cases 3 and 4 after infection with lentivirus Cas9 or lentivirus Cas9 plus lentivirus gRNAs A and B. Cells were treated with anti-CD2, CD3, and CD28 covered beads (Miltenyi Biotec) at the cells:bead ratio of 2:21 or PMA/TSA cocktail (PMA 25 nM/TSA 250 nM) for 48 hours, then counted and Gag p24 in supernatants was measured. **C.**

Figure S13. Amplification plots and standard curves used for absolute quantification of human beta-globin (A,B) and HIV-1 Gag (C,D) genes copy number in each sample. Serial dilutions of genomic DNA obtained from U1 monocytic cell line were prepared starting from 3.3 ug/ml which corresponds to 10^5 genome copies in 10ul/reaction and finishing at 0.33 ng/ml corresponding to 10 genome copies in 10 ul/reaction. U1 cells contain 2 single, full length copies of HIV-1 provirus

per genome, integrated in chromosome 2 and X, equal to beta-globin gene copies (2 per diploid genome).

Table S1: Mapping rate and coverage

Sample	+Cas9/+gRNA	+Cas9/-gRNA
Total	2304621804 (100%)	2153253838 (100%)
Duplicate	42615862 (19.60%)	326664344 (15.50%)
Mapped	2175107441 (94.38%)	2108094482 (97.90%)
Properly mapped	2133746358 (92.59%)	2057204364 (95.54%)
PE mapped	2173896582 (94.33%)	2107021448 (97.85%)
SE mapped	2421718 (0.11%)	2146068 (01.10%)
With mate mapped to a different chr	930716 (0.41%)	9569904 (0.33%)
With mate mapped to a different chr (mapQ>=5)	6944857 (0.30%)	7044381 (0.33%)
Average sequencing depth	112.72	109.25
Coverage	99.67%	99.69%
Coverage at least 4X	99.48%	99.51%
Coverage at least 10X	99.00%	99.08%
Coverage at least 20X	97.29%	97.57%

Total: The number of total clean rads
Duplicate: The number of duplication reads
Mapped: the number of total reads that mapped to the reference genome (percentage)
Properly mapped: The number of reads that mapped to the reference genome and the direction is right
PE mapped: The number of pair-end reads that mapped to the reference genome (percentage)
SE mapped: The number of single-end reads that mapped to the reference genome
With mate mapped to a different chr: The number of mate reads that mapped to the different chromosomes (percentage)
With mate mapped to a different chr (mapQ>=5): The number of mate reads that mapped to the different chromosomes and the MAQ>5
Average sequencing depth: The average sequencing depth that mapped to the reference genome
Coverage: The sequence coverage of the genome
Coverage at least 4X: The percentage of bases with depth >4X in whole genome bases
Coverage at least 10X: The percentage of bases with depth >10X in whole genome bases
Coverage at least 20X: The percentage of bases with depth >20X in whole genome bases

Table S2: Distribution of Insertion/Deletions (InDels) in different genomic regions

Sample	+Cas9/+gRNA	+Cas9/-gRNA	+Cas9/+gRNA over – +Cas9/-gRNA
CDS	1701	1746	164
frameshift_deletion	866	910	124
frameshift_insertion	279	275	33
nonframeshift_deletion	235	232	2
nonframeshift_insertion	187	196	0
stopgain	16	11	1
stoploss	1	1	0
unknown	117	121	4
Intronic	537492	538344	12229
UTR3	12154	12144	354
UTR5	1450	1446	58
Splicing	498	500	20
ncRNA_exonic	2638	2629	78
ncRNA_intronic	71426	71581	1758
ncRNA_UTR3	389	388	16
ncRNA_UTR5	45	50	2
ncRNA_splicing	80	74	1
upstream	9229	9256	209
downstream	10296	10204	199
intergenic	711001	712949	17310
Total	1358399	1361311	32399

Note Sample: Sample name

CDS: the number of InDel in exonic region

frameshift_deletion: a deletion of one or more nucleotides that cause frameshift changes in protein coding sequence. The deletion length is not multiple of 3

frameshift_insertion: an insertion of one or more nucleotides that cause frameshift changes in protein coding sequence. The insertion length is not multiple of 3.

nonframeshift_deletion: non-frameshift deletion, does not change coding protein frame deletion, the deletion length is multiple of 3.

nonframeshift_insertion: non-frameshift insertion, does not change coding protein frame deletion, the deletion length is multiple of 3.

stopgain: frameshift insertion/deletion, nonframeshift insertion/deletion or block substitution that lead to the immediate creation of stop codon at the variant site.

stoploss: frameshift insertion/deletion, nonframeshift insertion/deletion or block substitution that lead to the immediate elimination of stop codon at the variant site.

unknown: unknown function (due to various errors in the gene structure definition in the database file.

intronic: the number of InDel in intronic region

UTR3: the number of InDel in 3' UTR region

UTR5: the number of InDel in 5' UTR region

splicing: the number of InDel in 4bp splicing junction region

ncRNA_exonic: the number of InDel in non-coding RNA exonic region

ncRNA_intronic: the number of InDel in non-coding RNA intronic region

ncRNA_UTR3: the number of InDel in 3'UTR of non-coding RNA

ncRNA_UTR5: the number of InDel in 5'UTR of non-coding RNA

ncRNA_splicing: the number of InDel in 4bp splicing junction of non-coding RNA

upstream: the number of InDel in the 1 kb upstream region of transcription start site

downstream: the number of InDel in the 1 kb downstream region of transcription ending site

intergenic: the number of InDel in the intergenic region

Total: the total number of InDel

Table S3: Distribution of Single Nucleotide Polymorphisms (SNP) in different genomic regions

Sample	+Cas9/+gRNA	+Cas9/-gRNA	+Cas9/+gRNA over – +Cas9/-gRNA
CDS	29643	29982	1085
synonymous_SNP	13747	13894	347
missense_SNP	14924	15091	679
stopgain	378	394	43
stoploss	15	15	0
unknown	579	588	16
Intronic	1369993	1374507	17921
UTR3	28450	28628	564
UTR5	6626	6659	193
Splicing	845	882	32
ncRNA_exonic	12827	12918	175
ncRNA_intronic	206923	207516	2352
ncRNA_UTR3	809	824	11
ncRNA_UTR5	164	171	5
ncRNA_splicing	139	141	2
upstream	26386	26577	493
downstream	25707	25782	403
intergenic	2252883	2258511	23378
Total	3961395	3973098	46614

Note Sample: Sample name
CDS: the number of Somatic SNP in exonic region
synonymous_SNP: a single nucleotide change that does not cause an amino acid change
missense_SNP: a single nucleotide change that causes an amino acid change
stopgain: a nonsynonymous SNP that leads to the immediate creation of stop codon at the variant site
stoploss: a nonsynonymous SNP that leads to the immediate elimination of stop codon at the variant site.
unknown: unknown function (due to various errors in the gene structure definition in the database file).
intronic: the number of Somatic SNP in intronic region
UTR3: the number of Somatic SNP in 3' UTR region
UTR5: the number of Somatic SNP in 5' UTR region
intergenic: the number of Somatic SNP in the intergenic region
ncRNA_exonic: the number of Somatic SNP in non-coding RNA exonic region
ncRNA_intronic: the number of Somatic SNP in non-coding RNA intronic region
upstream: the number of Somatic SNP in the 1 kb upstream region of transcription start site
downstream: the number of Somatic SNP in the 1 kb downstream region of transcription ending site
splicing: the number of Somatic SNP in 10bp splicing junction region
ncRNA_UTR3: the number of Somatic SNP in 3'UTR of non-coding RNA
ncRNA_UTR5: the number of Somatic SNP 5'UTR of non-coding RNA
ncRNA_splicing: the number of Somatic SNP in 10bp splicing junction of non-coding RNA
Total: the total number of Somatic SNP

Table S4: Summary of SVN, InDels and SVs

	Total InDels	Somatic InDels[a]	Post dbSNP Filter	Post Homopolymeric Filter
+Cas9/-gRNA	1361311			
+Cas9/+gRNA	1358399	32399	30156	989

	Total SNVs	Somatic SNVs[b]	Post dbSNP Filter
+Cas9/-gRNA	3973098		
+Cas9/+gRNA	3961395	46614	43848

	Total SVs[c]	Somatic SVs[d]
+Cas9/-gRNA	3433	
+Cas9/+gRNA	3487	52

[a]Somatic InDels – means the specific InDels in +Cas9/+gRNA compared to control cell lines called by Strelka.
[b]Somatic SNVs – means the specific SNVs in +Cas9/+gRNA compared to control cell lines called by MuTect.
[c]Total SV – only includes the SV types of deletion and insertion called by Crest
[d]Somatic SVs – means the specific SVs (deletion and insertion) in +Cas9/+gRNA compared to control cell lines called by Crest

Figure S2

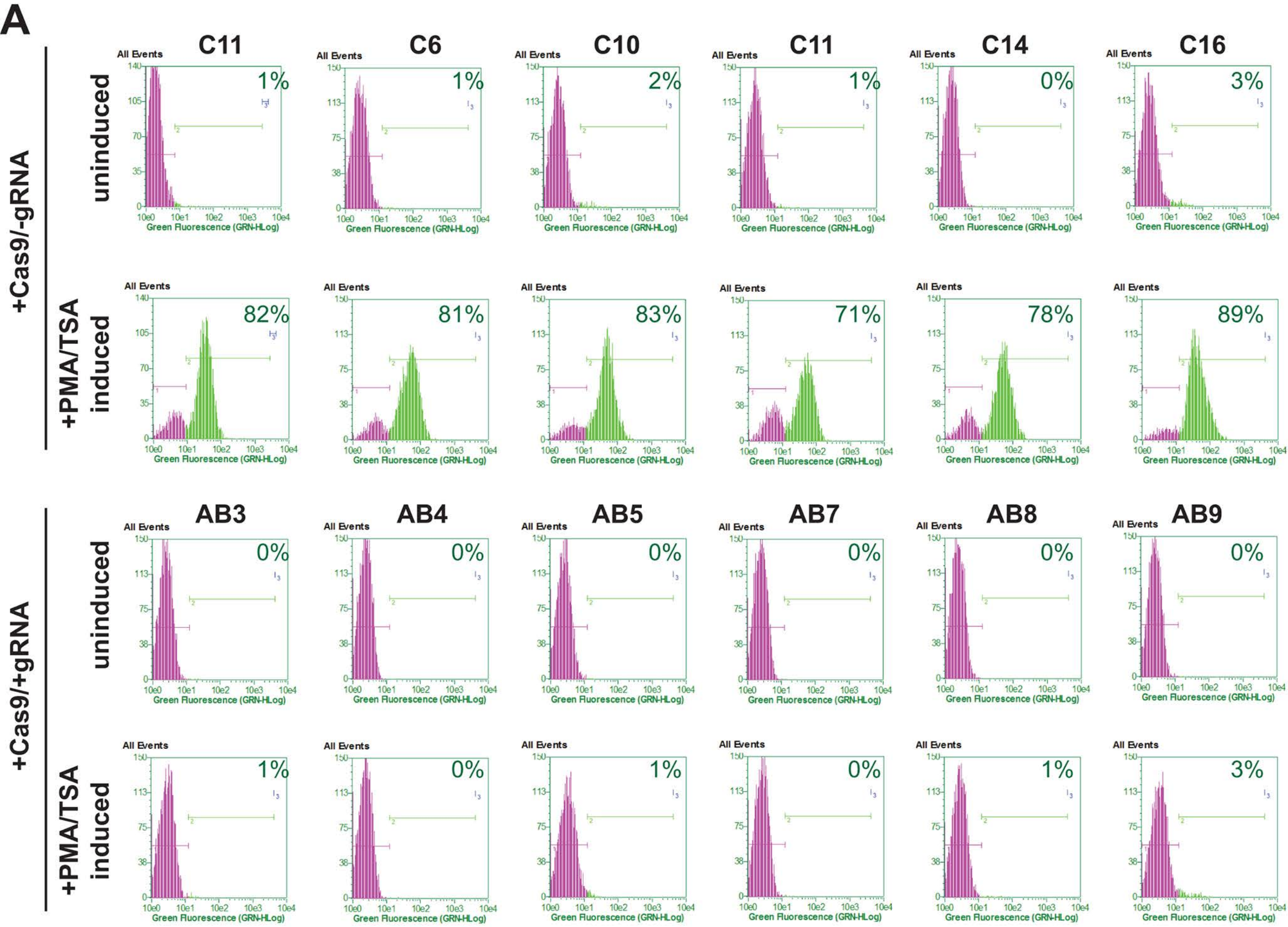

A
+Cas9/-gRNA
uninduced
+PMA/TSA induced
C11
C6
C10
C11
C14
C16
1%
1%
2%
1%
0%
3%
82%
81%
83%
71%
78%
89%
+Cas9/+gRNA
AB3
AB4
AB5
AB7
AB8
AB9
0%
0%
0%
0%
0%
0%
1%
0%
1%
0%
1%
3%
All Events
Green Fluorescence (GRN-HLog)

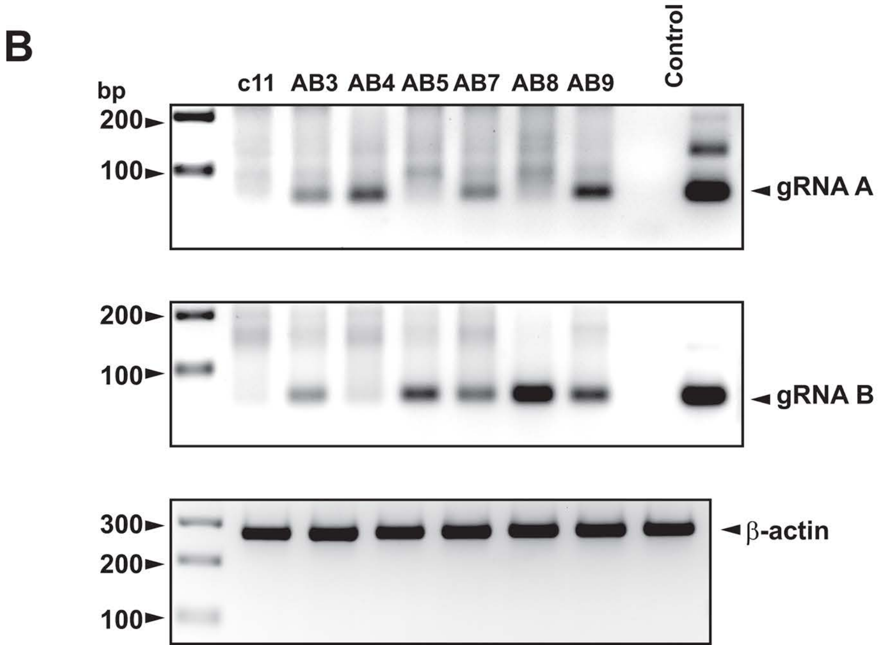

B
bp
c11
AB3
AB4
AB5
AB7
AB8
AB9
Control
200
100
gRNA A
gRNA B
300
β-actin

Figure S3

A

	Chromosome	Start	End	Gene Name	Functional Region	Type	
HIV-1 Insertion One	1	114338320	1143383420	RSBN1	intronic	CTX	CACCAGAGCACAGGACTCATTCAACAGTCCTCAATATTCTTATGTGGTTGAAGTGTT<br>TAATATCTAATTTAATCATAATCTGAAATGTTCTTAAAAAGTGGTTATTTTTTAAAT<br>CTCAAAGTAAGTATCAAGGTAAGTGGAAGGGCTAATTCACTCCCAACGAAGACAAGA<br>TATCCTTGATCTGTGGATCTACCACACACAAGGCTACTTCCCTGATTGGCAGAACTA<br>CACACCAGGGCCAGGGATCAGATATCCACTGACCTTTGGATGGTGCTACAAGCTAG<br>:5'LTR
	ChrVirus	1	1	---	intergenic	CTX	
	ChrVirus	9709	9709	---	intergenic	CTX	3'LTR:TCAAGTAGTGTGTGCCCGTCTGTTGTGTGACTCTGGTAACTAGAGATCCCT<br>CAGACCCTTTTAGTCAGTGTGGAAAATCTCTAGCAGTAAGTTGTTGGTACAGTCATT<br>TAAATTTCAGCTCCATTTTAAAAAATTAATTGGAGGACAAAAATCAGCAGGGAATAT<br>TTCAAGATATTTTCTTTTATTAACTATTTAGACTGGATACTGTCTGTTGACATACAA<br>ATTTGATC
	1	114338815	114338315	RSBN1	intronic	CTX	
HIV-1 Insertion Two	16	1991378	1991378	MSRB1	exonic	CTX	CACAGCCTACCCTCGGAACGGGGGCAGCGCTGTCTTTGCCTGGGTTGGTGGATTTGG<br>GAGCTTGACCCCGGAAAGGCGGGAGCTGATGACTCCACATTTGCCTCTCCTTCCACC<br>ACAGGCGTTTACGTGTGTGCCAAGTGTGGCTATTGGAAGGGCTAATTCACTCCCAAC<br>GAAGACAAGATATCCTTGATCTGTGGATCTACCACACACAAGGCTACTTCCCTGATT<br>GGCAGAACTACACACCAGGGCCAGGGATCAGATATCCACTGACCTTTGGATGGTGCT<br>ACAAGCTAG:5'LTR
	ChrVirus	1	1	---	intergenic	CTX	
	ChrVirus	9709	9709	---	intergenic	CTX	3'LTR:AAGTAGTGTGTGCCCGTCTGTTGTGTGACTCTGGTAACTAGAGATCCCTCA<br>GACCCTTTTAGTCAGTGTGGAAAATCTCTAGCAGTGCTATGAGCTGTTCTCCAGCCG<br>CTCGAAGTATGCACACTCGTCTCCATGGCCGGCGTTCACCGAGACCATTCACGCCGA<br>CAGCGTGGCCAAGCGTCCGGAGCACAATAGATCTGAAGCCTTGAAGG
	16	1991382	1991382	---	exonic	CTX	

B

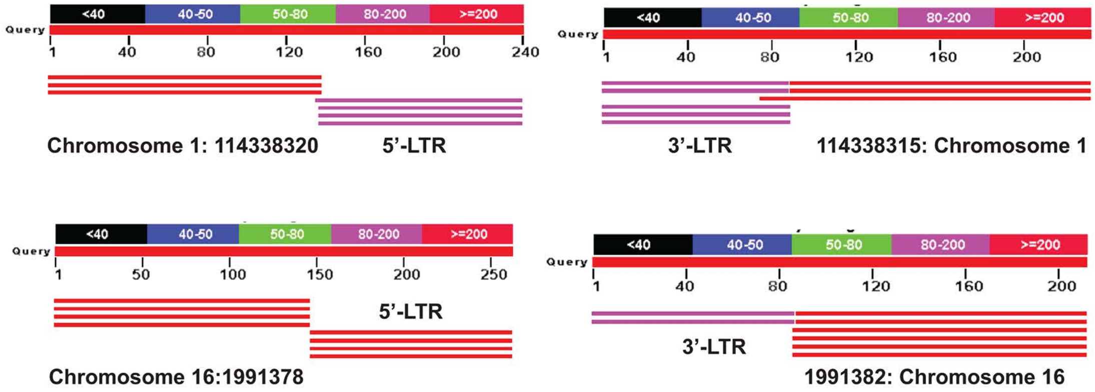

Figure S4

A

CH1 RSNB1LTR/NL4-3 LTRs sequences alignment

```
                RSNB1+16911
NL4-3LTR5'      ------------------------------------------------------------
CH1RSNB1LTR     GAGCACAGGACTCATTCAACAGTCCTCAATATTCTTATGTGGTTGAAGTGTTTAATATCT
NL4-3LTR3'      ------------------------------------------------------------

NL4-3LTR5'      ------------------------------------------------------------
CH1RSNB1LTR     AATTTAATCATAATCTGAAATGTTCTTAAAAAGTGGTTATTTTTTAAATCTCAAAGTAAG
NL4-3LTR3'      ------------------------------------------------------------

                 RSNB1+16780/LTR-454
NL4-3LTR5'      ------------TGGAAGGGCTAATTTGGTCCCAAAAAAGACAAGAGATCCTTGATCTGT
CH1RSNB1LTR     TATCAAGGTAAGTGGAAGGGCTAATTCACTCCCAACGAAGACAAGATATCCTTGATCTGT
NL4-3LTR3'      ------------TGGAAGGGCTAATTCACTCCCAAAGAAGACAAGATATCCTTGATCTGT
                            **************   ******  *********  ************

NL4-3LTR5'      GGATCTACCACACACAAGGCTACTTCCCTGATTGGCAGAACTACACACCAGGGCCAGGGA
CH1RSNB1LTR     GGATCTACCACACACAAGGCTACTTCCCTGATTGGCAGAACTACACACCAGGGCCAGGGA
NL4-3LTR3'      GGATCTACCACACACAAGGCTACTTCCCTGATTGGCAGAACTACACACCAGGGCCAGGGG
                *********************************************************** 

                   LTR A target     PAM
NL4-3LTR5'      TCAGATATCCACTGACCTTTGGATGGTGCTTCAAGTTAGTACCAGTTGAACCAGAGCAAG
CH1RSNB1LTR     TCAGATATCCACTGACCTTTGGATGGTGCTACAAGCTAGTACCAGTTGAGCAAGAGAAGG
NL4-3LTR3'      TCAGATATCCACTGACCTTTGGATGGTGCTACAAGCTAGTACCAGTTGAGCCAGATAAGG
                ****************************** **** ************* * ***  * *

NL4-3LTR5'      TAGAAGAGGCCAAATAAGGAGAGAAGAACAGCTTGTTACACCCTATGAGCCAGCATGGGA
CH1RSNB1LTR     TAGAAGAAGCCAATGAAGGAGAGAACACCCGCTTGTTACACCCTGTGAGCCTGCATGGGA
NL4-3LTR3'      TAGAAGAGGCCAATAAAGGAGAGAACACCAGCTTGTTACACCCTGTGAGCCTGCATGGAA
                ******* *****  ********** * * ************** ****** ****** *

NL4-3LTR5'      TGGAGGACCCGGAGGGAGAAGTATTAGTGTGGAAGTTTGACAGCCTCCTAGCATTTCGTC
CH1RSNB1LTR     TGGATGACCCGGAGAGAGAAGTATTAGAGTGGAGGTTTGACAGCCGCCTAGCATTTCATC
NL4-3LTR3'      TGGATGACCCTGAGAGAGAAGTGTTAGAGTGGAGGTTTGACAGCCGCCTAGCATTTCATC
                **** ***** *** ******* **** ***** ********** ********** **

                                    PAM INSERTION     LTR B target
NL4-3LTR5'      ACATGGCCCGAGAGCTGCATCCGGAG-------TACTACAAAGACTGCTGACATCGAGCT
CH1RSNB1LTR     ACATGGCCCGAGAGCTGCATCCGGAGCTAAGTTTACTTCAAGAACTGCTGACATCGAGCT
NL4-3LTR3'      ACGTGGCCCGAGAGCTGCATCCGGAG-------TACTTCAAGAACTGCTGACATCGAGCT
                ** ***********************       **** ***  ****************

NL4-3LTR5'      TTCTACAAGGGACTTTCCGCTGGGGACTTTCCAGGGAGGTGTGGCCTGGGCGGGACTGGG
CH1RSNB1LTR     TGCTACAAGGGACTTTCCGCTGGGGACTTTCCAGGGAGGCGTGGCCTGGGCGGGACTGGG
NL4-3LTR3'      TGCTACAAGGGACTTTCCGCTGGGGACTTTCCAGGGAGGCGTGGCCTGGGCGGGACTGGG
                * ************************************* ********************

NL4-3LTR5'      GAGTGGCGAGCCCTCAGATGCTACATATAAGCAGCTGCTTTTTGCCTGTACTGGGTCTCT
CH1RSNB1LTR     GAGTGGCGAGCCCTCAGATGCTGCATATAAGCAGCTGCTTTTTGCTTGTACTGGGTCTCT
NL4-3LTR3'      GAGTGGCGAGCCCTCAGATGCTGCATATAAGCAGCTGCTTTTTGCCTGTACTGGGTCTCT
                ********************** ********************** **************

NL4-3LTR5'      CTGGTTAGACCAGATCTGAGCCTGGGAGCTCTCTGGCTAACTAGGGAACCCACTGCTTAA
CH1RSNB1LTR     CTGGTTAGACCAGATCTGAGCCTGGGAGCTCTCTGGCTAACTAGGGAACCCACTGCTTAA
NL4-3LTR3'      CTGGTTAGACCAGATCTGAGCCTGGGAGCTCTCTGGCTAACTAGGGAACCCACTGCTTAA
                ************************************************************

NL4-3LTR5'      GCCTCAATAAAGCTTGCCTTGAGTGCTCAAAGTAGTGTGTGCCCGTCTGTTGTGTGACTC
CH1RSNB1LTR     GCCTCAATAAAGCTTGCCTTGAGTGCTTCAAGTAGTGTGTGCCCGTCTGTTGTGTGACTC
NL4-3LTR3'      GCCTCAATAAAGCTTG-CTTGAGTGCTTCAAGTAGTGTGTGCCCGTCTGTTGTGTGACTC
                **************** **********  *******************************
                                                  LTR+181/RSNB1+16779(duplication)
NL4-3LTR5'      TGGTAACTAGAGATCCCTCAGACCCTTTTAGTCAGTGTGGAAAATCTCTAGCAG------
CH1RSNB1LTR     TGGTAACTAGAGATCCCTCAGANCCTTTTAGTCAGTGTGGAAAATCTCTAGCAGTAAGTT
NL4-3LTR3'      TGGTAACTAGAGATCCCTCAGACCCTTTTAGTCAGTGTGGAAAATCTCTAGCAC------
                ********************** *******************************

NL4-3LTR5'      ------------------------------------------------------------
CH1RSNB1LTR     GTTGGTACAGTCATTTAAATTTCAGCTCCATTTTAAAAAATTAATTGGNNGACAAAAATC
NL4-3LTR3'      ------------------------------------------------------------

                                                             RSNB1+16648
NL4-3LTR5'      ------------------------------------------------------------
CH1RSNB1LTR     AGCAGGAATATTTCAAGATATTTTCTTTTATTAACTATTTAGACTGGATACTGNCTGTTG
NL4-3LTR3'      ------------------------------------------------------------
```

B

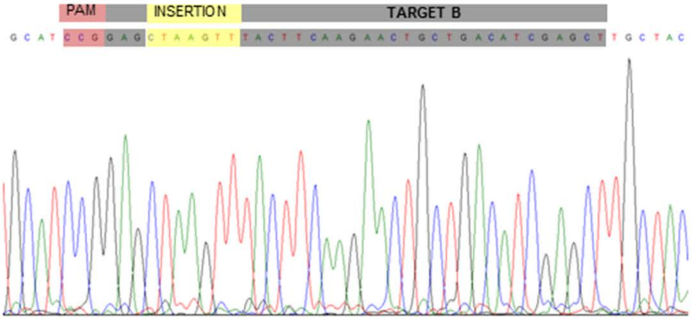

Figure S5

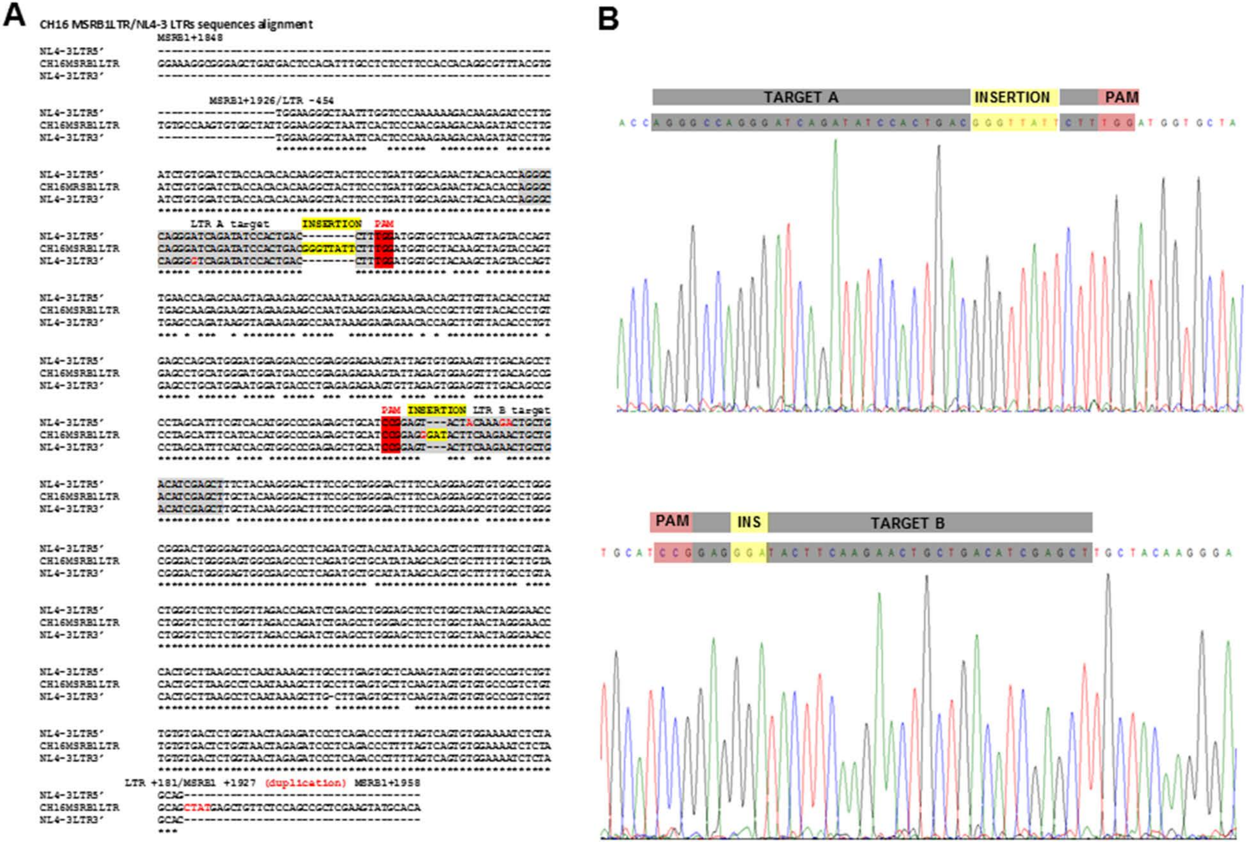
A
CH16 MSRB1LTR/NL4-3 LTRs sequences alignment
MSRB1+1848
NL4-3LTR5'
CH16MSRB1LTR
NL4-3LTR3'
MSRB1+1926/LTR -454
LTR A target
INSERTION
PAM
LTR B target
LTR +181/MSRB1 +1927 (duplication) MSRB1+1958
B
TARGET A
INSERTION
PAM
INS
TARGET B

Figure S6

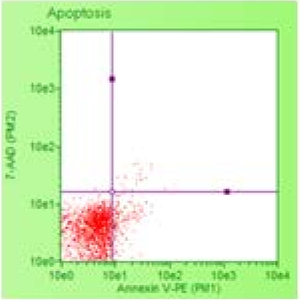

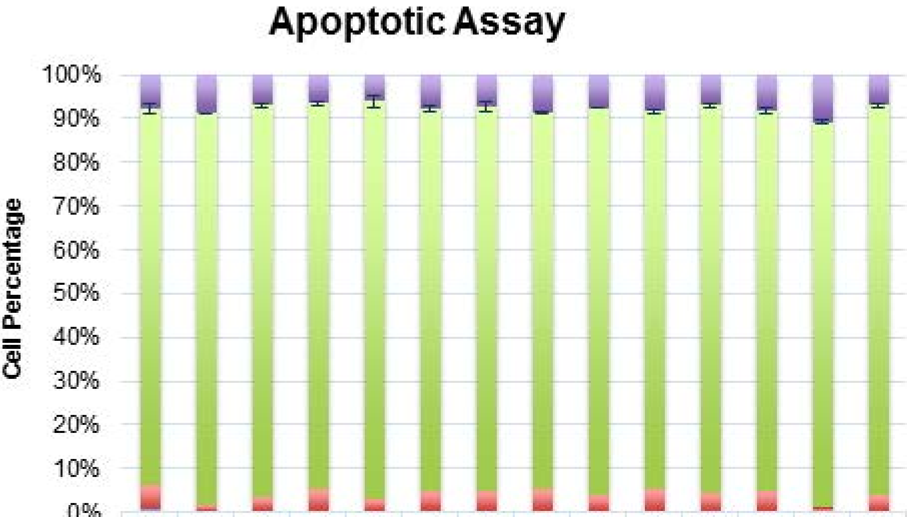

	C1	C2	C3	C4	C5	C6	C7	C8	C9	C10	C11	C12	C13	C14
Annexin(+) 7-AAD(-)	7.70	8.80	6.95	6.55	6.00	7.55	7.35	8.70	7.50	8.35	7.00	8.15	10.75	6.95
Annexin(-) 7-AAD(-)	85.85	89.65	89.40	88.15	91.05	87.60	87.85	85.95	88.65	86.55	88.45	87.15	88.15	89.00
Annexin(+) 7-AAD(+)	5.90	1.55	3.40	5.05	2.85	4.75	4.60	5.10	3.65	4.90	4.50	4.50	1.10	3.95
Annexin(-) 7-AAD(+)	0.55	0.05	0.25	0.30	0.15	0.15	0.20	0.35	0.25	0.25	0.10	0.20	0.05	0.15

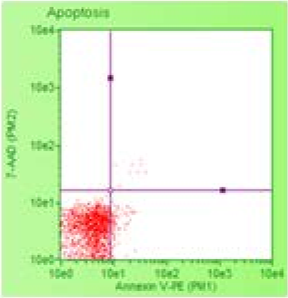

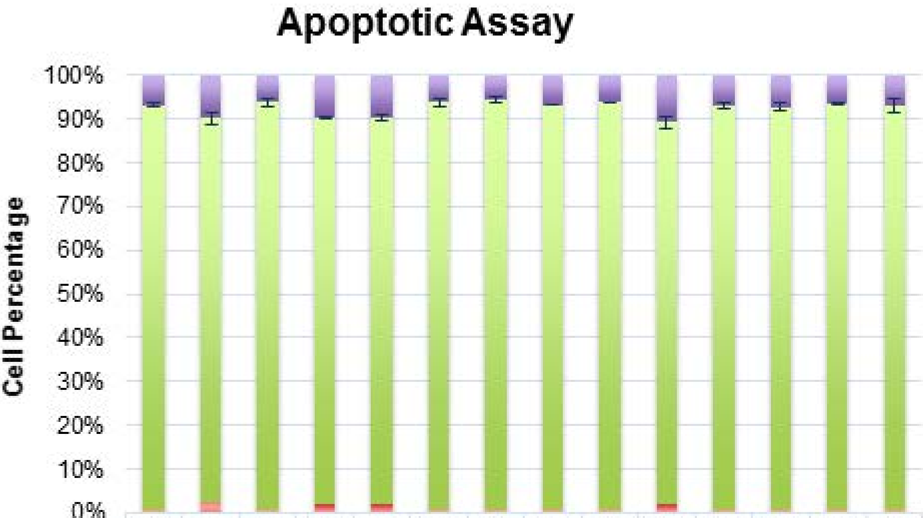

	AB1	AB2	AB3	AB4	AB5	AB6	AB7	AB8	AB9	AB10	AB11	AB12	AB13	AB14
Annexin(+) 7-AAD(-)	6.75	9.70	6.15	9.85	9.70	6.20	5.50	6.85	6.10	10.75	6.80	7.25	6.55	6.95
Annexin(-) 7-AAD(-)	92.55	87.85	93.35	87.95	88.25	93.05	93.80	92.60	93.35	87.10	92.70	92.05	92.95	92.45
Annexin(+) 7-AAD(+)	0.70	2.40	0.50	1.90	1.90	0.75	0.60	0.35	0.50	2.05	0.50	0.70	0.50	0.55
Annexin(-) 7-AAD(+)	0.00	0.10	0.05	0.30	0.15	0.05	0.15	0.10	0.10	0.15	0.00	0.05	0.05	0.05

Figure S7

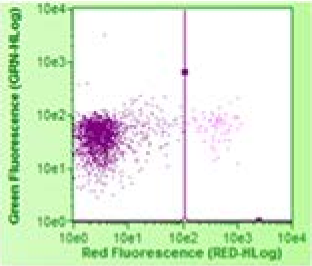

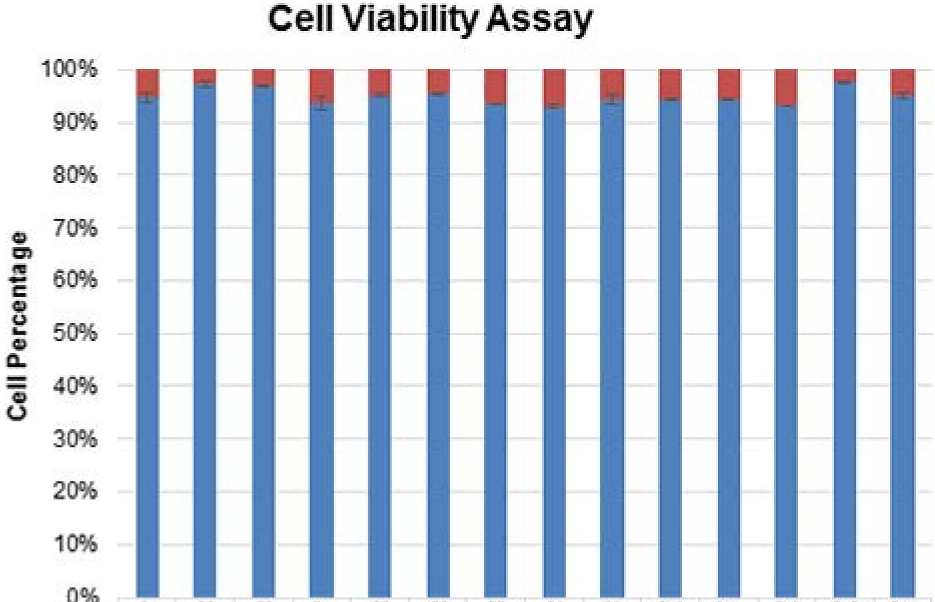

	C1	C2	C3	C4	C5	C6	C7	C8	C9	C10	C11	C12	C13	C14
Dead Cell Percentage	5.24	2.78	3.12	6.24	4.88	4.60	6.46	6.90	5.66	5.66	5.56	6.88	2.32	4.82
Live Cell Percentage	94.76	97.22	96.88	93.76	95.12	95.40	93.54	93.10	95.20	94.12	94.44	93.12	97.68	95.18

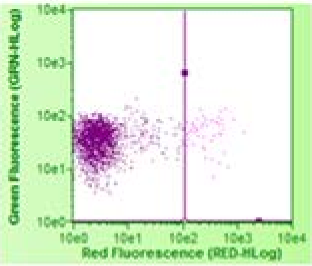

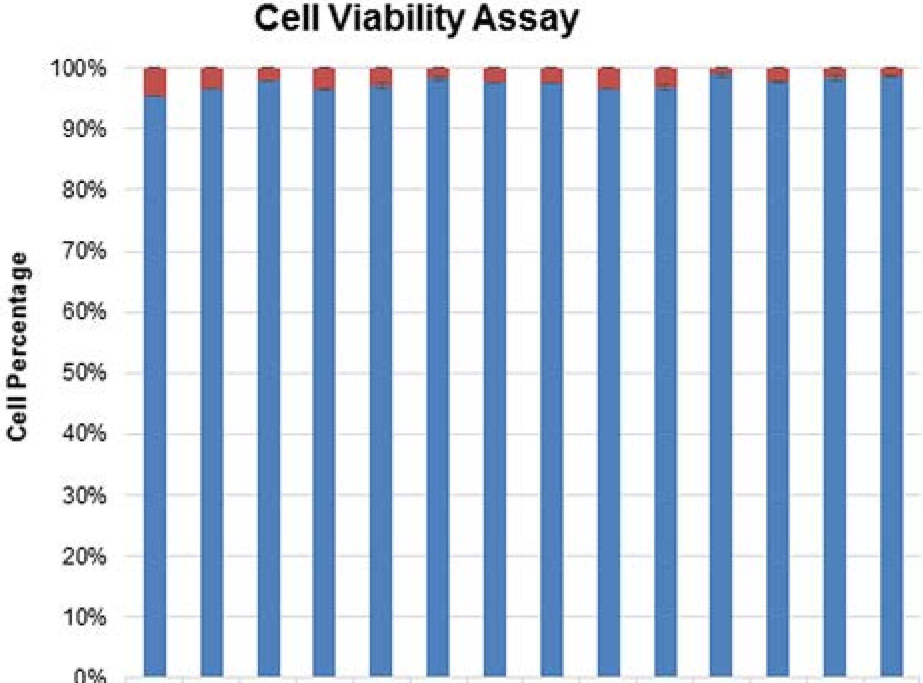

	AB1	AB2	AB3	AB4	AB5	AB6	AB7	AB8	AB9	AB10	AB11	AB12	AB13	AB14
Dead Cell Percentage	4.66	3.42	2.10	3.44	2.82	1.68	2.40	2.46	3.38	3.20	1.16	2.28	1.60	1.28
Live Cell Percentage	95.34	96.58	97.90	96.56	97.18	98.32	97.60	97.54	96.62	96.80	98.84	97.72	98.40	98.72

Figure S8

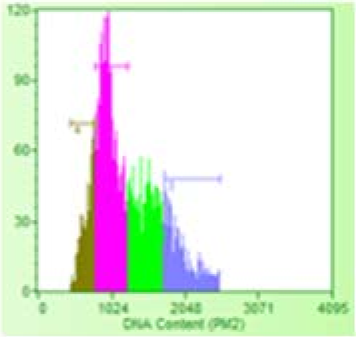

Cell Cycle Controls

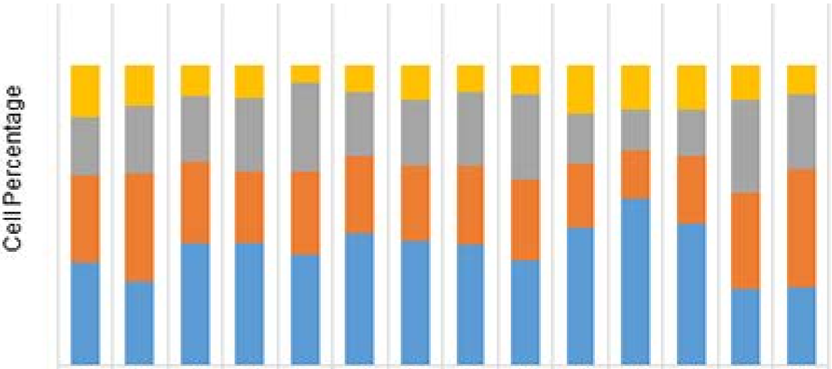

	C1	C2	C3	C4	C5	C6	C7	C8	C9	C10	C11	C12	C13	C14
Sub G0	17.7	13.5	10.8	11	5.8	9.3	11.7	9.1	9.9	16.4	14.6	15	11.7	9.7
G2/ M Phase	19.2	22.9	21.3	24.8	29.6	21.3	21.9	24.4	27.9	16.6	13.6	15.3	30.9	25.2
S Phase	28.7	35.7	27.2	23.8	28	25.7	25.5	26.6	27	21	16.1	22.6	32.4	39.5
G0/G1	34.4	28	40.8	40.4	36.6	43.6	41	39.9	35.1	45.9	55.6	47.1	25	25.6

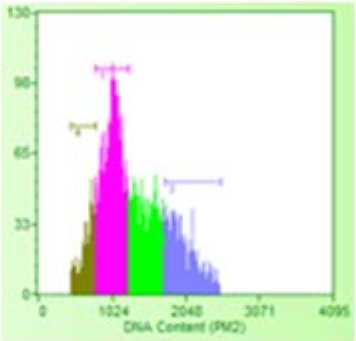

Cell Cycle Eradicated Clones

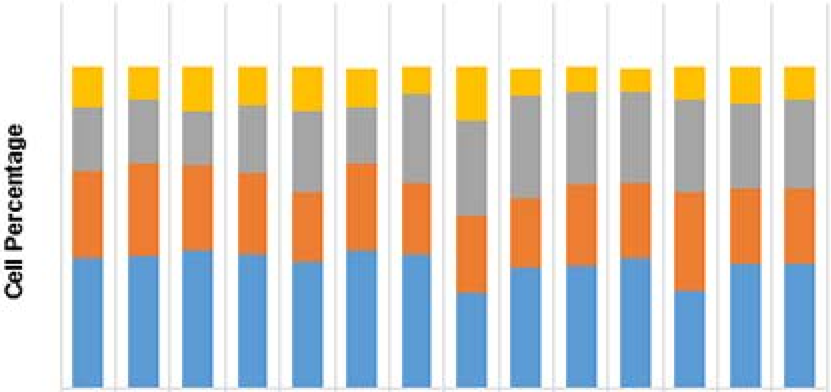

	AB1	AB2	AB3	AB4	AB5	AB6	AB7	AB8	AB9	AB10	AB11	AB12	AB13	AB14
Sub G0	12.6	10.2	13.3	11.5	13.7	12.5	8.1	16.6	8.7	7.3	7.4	10.1	11	10.1
G2/ M Phase	19.5	19.4	16.9	21.5	25.1	17.5	27.7	29.4	31.8	29.3	28.6	28.9	26.5	27.4
S Phase	27.4	29	26.6	25.1	21.9	26.5	22.2	24.4	21.7	25.4	23.2	30.6	23.6	23.7
G0/G1	40.5	41.4	43.2	41.9	39.3	43.4	42.1	29.6	37.7	38	40.7	30.5	38.9	38.9

Figure S9

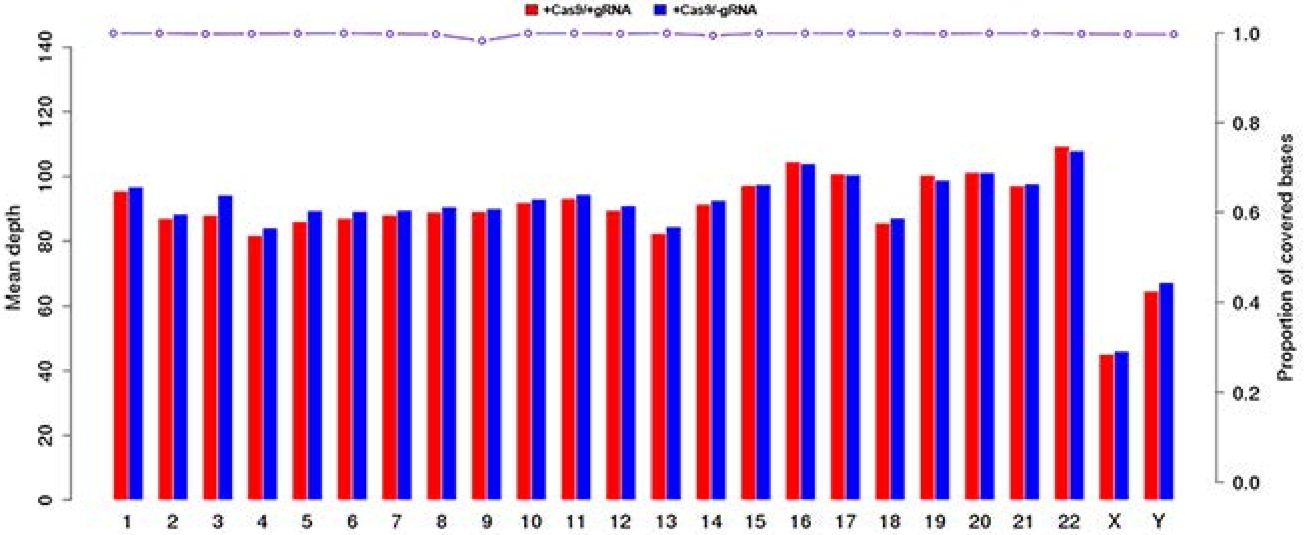

+Cas9/+gRNA
+Cas9/-gRNA
Mean depth
Proportion of covered bases
0
20
40
60
80
100
120
140
0.0
0.2
0.4
0.6
0.8
1.0
1
2
3
4
5
6
7
8
9
10
11
12
13
14
15
16
17
18
19
20
21
22
X
Y

Figure S10

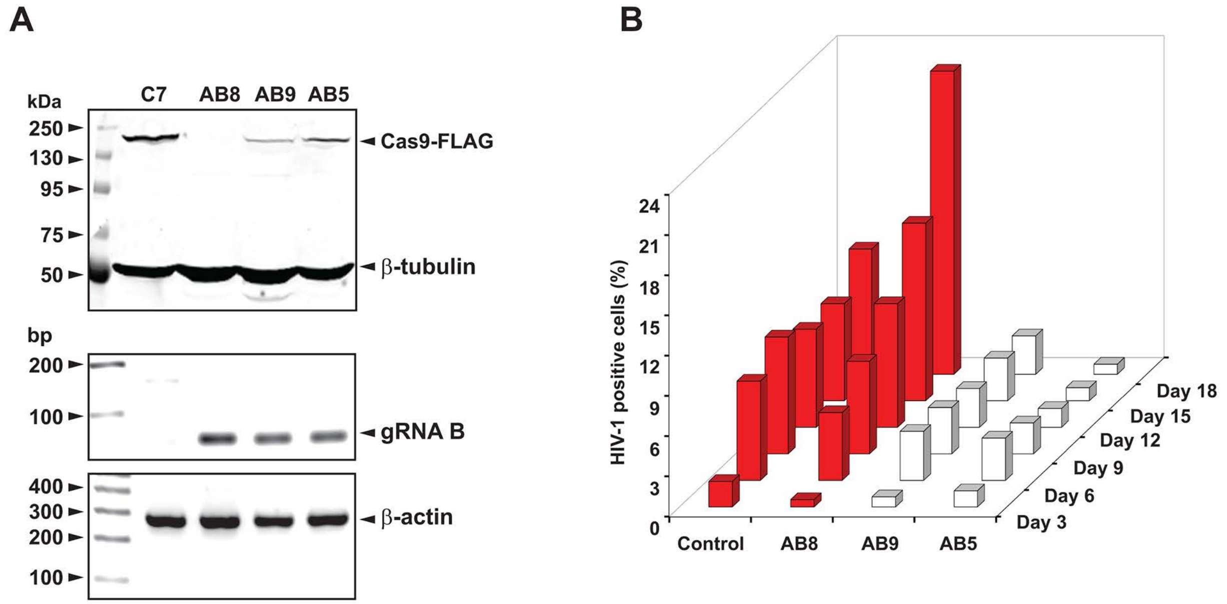

C

% HIV-1 positive cells

Days	C7	AB8	AB9	AB5
**3**	1.9 (± 0.71)	0.6 (± 0.21)	0.8 (± 0.07)	1.2 (± 0.42)
**6**	7.4 (± 1.41)	5.1 (± 0.64)	3.7 (± 0.92)	3.2 (± 0.21)
**9**	8.7 (± 0.28)	6.9 (± 0.42)	3.5 (± 0.64)	2.3 (± 0.14)
**12**	7.3 (± 0.14)	9.2 (± 0.57)	2.9 (± 0.99)	1.4 (± 1.13)
**15**	7.2 (± 0.11)	13.2 (± 0.30)	3.2 (± 0.14)	1.0 (± 0.20)
**18**	9.4 (± 0.21)	22.6 (± 1.70)	2.9 (± 0.07)	0.8 (± 0.07)

Figure S11

A

	Age/Gender	Ethnicity	VL	CD4	CD4%	ART
**Case 1 (TUR0001)**	43/M	AA	12.45	716	11	Atazanavir, Ritonavir, Raltegravir
**Case 2 (TUR0002)**	50/M	AA	<20.0	630	30	Ritonavir, Daruravir, Maravirus, Dalutegravir
**Case 3 (TUR0003)**	44/F	His	<20.0	714	39	Tenofovir/emtricitabine, Atazanavir, Ritonavir
**Case 4 (TUR0004)**	36/M	AA	523	16	8	Lamivudine, Ritonavir, Raltegravir, Darunavir

B

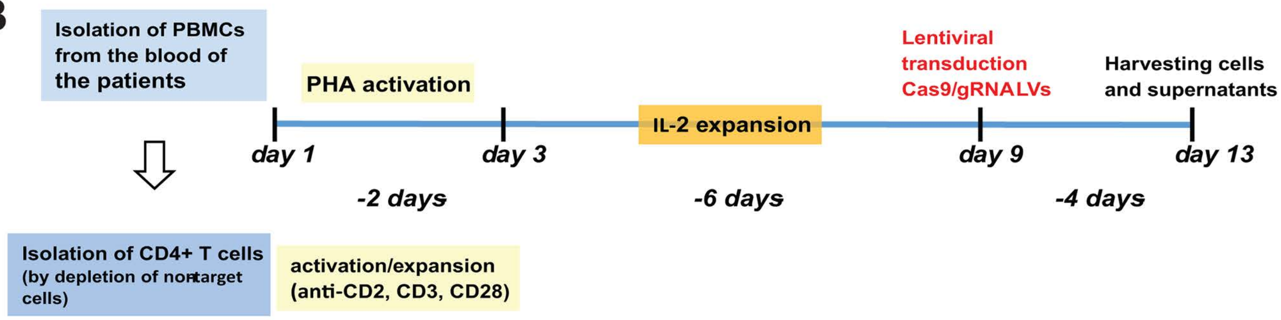

C

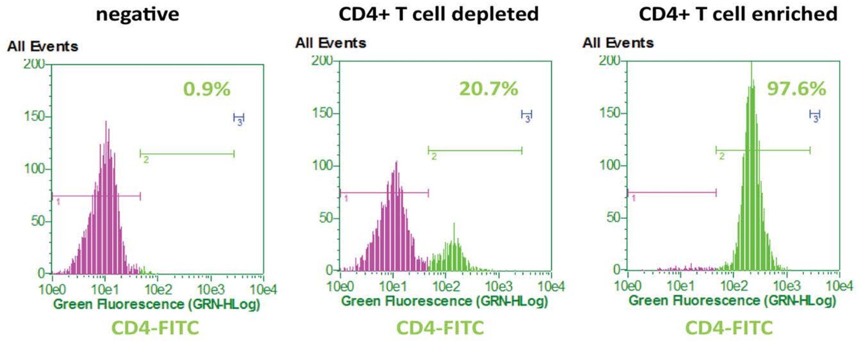

Figure S12

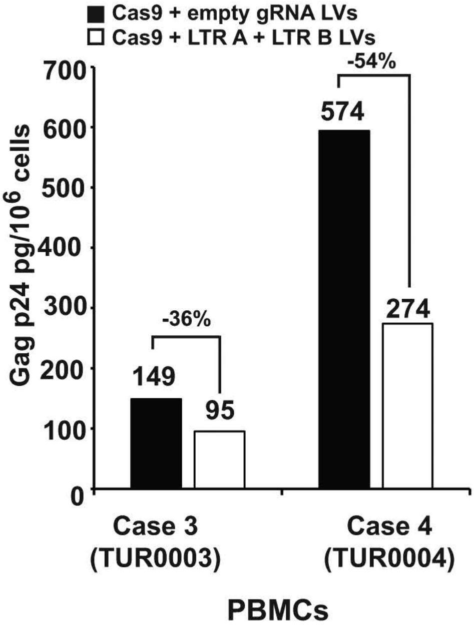
Cas9 + empty gRNA LVs
Cas9 + LTR A + LTR B LVs
Gag p24 pg/10^6 cells
700
600
500
400
300
200
100
0
-36%
149
95
-54%
574
274
Case 3
(TUR0003)
Case 4
(TUR0004)
PBMCs

Figure S13

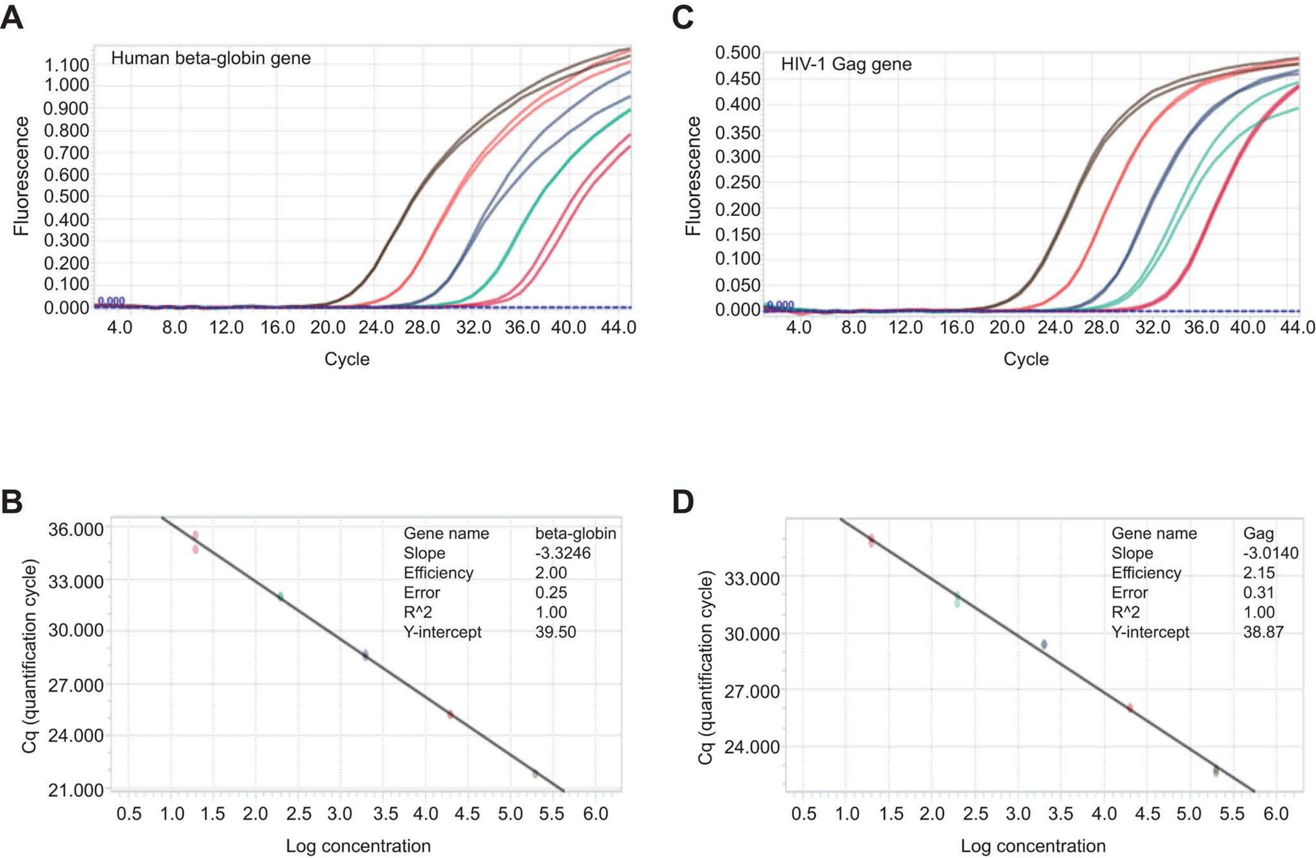
A
Human beta-globin gene
Fluorescence
1.100
1.000
0.900
0.800
0.700
0.600
0.500
0.400
0.300
0.200
0.100
0.000
4.0
8.0
12.0
16.0
20.0
24.0
28.0
32.0
36.0
40.0
44.0
Cycle
C
HIV-1 Gag gene
Fluorescence
0.500
0.450
0.400
0.350
0.300
0.250
0.200
0.150
0.100
0.050
0.000
Cycle
B
Cq (quantification cycle)
36.000
33.000
30.000
27.000
24.000
21.000
Gene name beta-globin
Slope -3.3246
Efficiency 2.00
Error 0.25
R^2 1.00
Y-intercept 39.50
0.5
1.0
1.5
2.0
2.5
3.0
3.5
4.0
4.5
5.0
5.5
6.0
Log concentration
D
Cq (quantification cycle)
33.000
30.000
27.000
24.000
Gene name Gag
Slope -3.0140
Efficiency 2.15
Error 0.31
R^2 1.00
Y-intercept 38.87
Log concentration
